# Identification of nocamycin biosynthetic gene cluster from *Saccharothrix syringae* NRRL B-16468 and generation of new nocamycin derivatives by manipulating gene cluster

**DOI:** 10.1186/s12934-017-0718-5

**Published:** 2017-06-09

**Authors:** Xuhua Mo, Chunrong Shi, Chun Gui, Yanjiao Zhang, Jianhua Ju, Qingji Wang

**Affiliations:** 1 Shandong Key Laboratory of Applied Mycology, School of Life Sciences, Qingdao Agricultural University, Qingdao, 266109 China; 2 CAS Key Laboratory of Tropical Marine Bio-resources and Ecology, Guangdong Key Laboratory of Marine Materia Medica, RNAM Center for Marine Microbiology, South China Sea Institute of Oceanology, Chinese Academy of Sciences, 164 West Xingang Rd., Guangzhou, 510301 China

**Keywords:** Nocamycins, Biosynthetic gene cluster, Cytochrome P450 oxidase, Post-tailoring modification, *Saccharothrix syringae*

## Abstract

**Background:**

Nocamycins I and II, produced by the rare actinomycete *Saccharothrix syringae*, belong to the tetramic acid family natural products. Nocamycins show potent antimicrobial activity and they hold great potential for antibacterial agent design. However, up to now, little is known about the exact biosynthetic mechanism of nocamycin.

**Results:**

In this report, we identified the gene cluster responsible for nocamycin biosynthesis from *S. syringae* and generated new nocamycin derivatives by manipulating its gene cluster. The biosynthetic gene cluster for nocamycin contains a 61 kb DNA locus, consisting of 21 open reading frames (ORFs). Five type I polyketide synthases (NcmAI, NcmAII, NcmAIII, NcmAIV, NcmAV) and a non-ribosomal peptide synthetase (NcmB) are proposed to be involved in synthesis of the backbone structure, a Dieckmann cyclase NcmC catalyze the releasing of linear chain and the formation of tetramic acid moiety, five enzymes (NcmEDGOP) are related to post-tailoring steps, and five enzymes (NcmNJKIM) function as regulators. Targeted inactivation of *ncmB* led to nocamycin production being completely abolished, which demonstrates that this gene cluster is involved in nocamycin biosynthesis. To generate new nocamycin derivatives, the gene *ncmG*, encoding for a cytochrome P450 oxidase, was inactivated. Two new nocamycin derivatives nocamycin III and nocamycin IV were isolated from the *ncmG* deletion mutant strain and their structures were elucidated by spectroscopic data analyses. Based on bioinformatics analysis and new derivatives isolated from gene inactivation mutant strains, a biosynthetic pathway of nocamycins was proposed.

**Conclusion:**

These findings provide the basis for further understanding of nocamycin biosynthetic mechanism, and set the stage to rationally engineer new nocamycin derivatives via combinatorial biosynthesis strategy.

**Electronic supplementary material:**

The online version of this article (doi:10.1186/s12934-017-0718-5) contains supplementary material, which is available to authorized users.

## Background

**Fig. 1:**
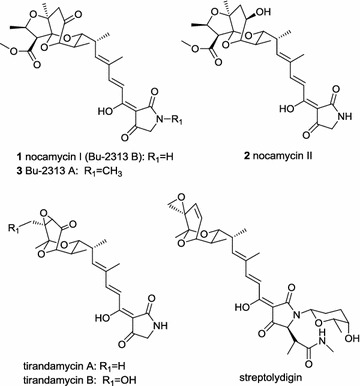
Structure of nocamycins and related compounds

Nocamycins I and II (Fig. 1), isolated from the broth of *Saccharothrix syringae* NRRL B-16468 by Russian scientists in 1977, belong to the tetramic acid (2, 4-pyrrolidinedione) family natural products [1–3]. The original structural assignment of nocamycin I was incorrect and it was revised by a Japanese research group [4]. The Japanese research group reported two compounds Bu-2313A and Bu-2313B from the strain *Microtetraspora caesia* ATCC 31295 nearly at the same time [5]. Further structural elucidation showed that Bu-2313B was virtually identical to nocamycin I [4]. Beyond the common tetramic acid structure, a tricyclic ketal structure is another interesting motif in nocamycins. In terms of structural viewpoint, streptolydigin, tirandamycins and tirandalydigin are closely related to nocamycins (Fig. 1). Among these compounds, nocamycins I and II are unique because they have a fused oxolane ring system other than an oxirane (spiro or fused) ring in streptolydigin, tirandamycin and tirandalydigin.

Nocamycin I (Bu-2313B) displays broad antimicrobial activity toward a panel of Gram-positive and Gram-negative anaerobic bacteria as well as some aerobic bacteria. Inhibitions of anaerobic bacteria *Bacteroides fragilis*, *Clostridium* sp., *Fusobacterium* sp., *Sphaerophorus* sp. by nocamycins are particularly potent, and the minimum inhibitory concentrations (MICs) are in the range of 0.1–0.4 μg/mL [5–7]. Further in vivo experiments conducted in mice showed that nocamycin I is effective in protecting mice against *B. fragilis* A20928-1 and *Clostridium perfingens* A9635 when administered by both oral and subcutaneous routes [5]. In addition, nocamycins show antitumor effects [1]. Up to now, the exact antibacterial mold of action of nocamycins has not been investigated. The closely related compounds tirandamycin and streptolydigin are validated to be inhibitors of bacterial RNA polymerase (RNAP), thus nocamycins are probably to be inhibitors of RNAP. In recent years, the molecular evidences for the structural basis of the RNAP interaction mechanism of this class of natural products have been disclosed by co-crystal complexes of streptolydigin with RNAPs from *Escherichia coli* and *Thermus thermophilus* [8, 9]. The key affinities of both bicyclic ketal and tetramic acid structures with RNAPs have been observed from the co-crystal complexes, indicating the substitution or modification in these two structural motifs is critical for the biological activity [8, 9]. Meanwhile, results of antibacterial activities of streptolydigin, tirandamycin and their congeners also demonstrated that the two featured motifs are closely related to the activity of this family of natural products [10–12].

The intriguing structure, action mold and biological activity of this small class of natural products attract more and more attentions from biochemists. So far, the gene clusters responsible for tirandamycin and streptolydigin biosynthesis have been identified from three different *Streptomyces* species by Sherman, Salas and Ju group, respectively [10, 13, 14]. Both tirandamycins and streptolydigin are assembled by hybrid iterative type I polyketide synthase (PKS) and non-ribosomal peptide synthase (NRPS). The functions of a number of genes related to post-tailoring, regulator and resistance involved in tirandamycin and streptolydigin biosynthetic pathway have been fully elucidated [12, 13, 15–20]. Some streptolydigin derivatives were generated by using combinatorial biosynthesis method [10]. In streptolydigin and tirandamycins biosynthetic pathway, a uniform strategy is employed to catalyze the formation of tetramic acid moiety [21]. The mechanism of bicyclic ketal structure formation remains unclear since no related gene candidates have been discovered in the two gene clusters. To fully understand the biosynthetic pathway of nocamycins, provide insights into the formation of bicyclic ketal structure and generate diversified nocamycin derivatives, we started to identify the nocamycin biosynthetic gene cluster from *S. syringae* NRRL B-16468. Here, we report the identification of nocamycin biosynthetic gene cluster and new nocamycin derivatives generated by manipulating the gene cluster.

## Methods

### Bacterial strains, plasmids, medium and culture conditions

The bacteria and plasmids used in this study are listed in Table 1. *S. syringae* was maintained on ISP4 agar medium. The medium used for fermentation of *S. syringae* and its mutant strains consists of 1% soybean, 3% glycerol, 0.5% mycose, 0.2% NaCl and 0.2% CaCO_3_. All cultures for *S. syringae* were incubated at 28 °C. For *E. coli*, Luria–Bertani (LB) liquid or agar media were used with appropriate antibiotics at a final concentration of: 100 μg/mL ampicillin (Amp), 50 μg/mL apramycin (Apr), 50 μg/mL kanamycin (Kan), 25 μg/mL chloramphenicol (Cml) and 50 μg/mL trimethoprim (TMP).

**Table 1:** Bacteria and plasmids used in this study

Strains or plasmids	Description	Reference or source
Strains		
*E. coli* LE392	Host strain of cosmid vector SuperCos I	Stratagene
*E. coli* DH5*α*	Host strain for general clone	Stratagene
*E. coli* ET12567/pUZ8002	Host strain for conjugation	[22]
*E. coli* BW25113	Host strain for PCR-targeting	[23]
*S. syringae*	Nocamycin-producing strain	NRRL
*S. syringae* MoS1001	*ncmB deletion mutant strains originated from Saccharothrix syringae*	This study
*S. syringae* MoS1002	*ncmL deletion mutant strains originated from Saccharothrix syringae*	This study
*S. syringae* MoS1003	*ncmG deletion mutant strains originated from Saccharothrix syringae*	This study
Plasmids		
SuperCosI	Amp^r^, Kan^r^, cosmid vector	Stratagene
pIJ790	Cml^r^, including λ-RED (*gam, bet, exo*) for PCR-targeting	[24]
pIJ773	Apr^r^, source of *acc(3)IV* and *oriT* fragment	[24]
pUZ8002	Kan^r^, including *tra* for conjugation	[25]
p5-C-9	Amp^r^, Kan^r^, harboring *ncmL*gene	This study
p2-H-12	Amp^r^, Kan^r^, harboring *ncmG* gene	This study

### DNA sequencing, assembly and analysis

After growing in TSB medium for 48–72 h, the genomic DNA of *S. syringae* NRRL B-16468 was extracted according to standard protocols [26]. Then, the genomic DNA was shotgun sequenced and annotated by Shanghai South Gene Technology Co. Ltd. (Shanghai, China). The gene cluster responsible for secondary metabolite biosynthesis was analyzed by antiSMASH online analysis tool (http://antismash.secondarymetabolites.org/). DNA and corresponding protein sequences in nocamycin gene cluster were analyzed by ORF finder program (http://www.ncbi.nlm.nih.gov/gorf/gorf.html), Frameplot 2.3.2 program (http://www.nih.go.jp/~jun/cgi-bin/frameplot.pl), and BLAST program (http://blast.ncbi.nlm.nih.gov/).

### Construction and screening of *S. syringae* genomic library

Genomic library of *S. syringae* NRRL B-16468 was constructed using SuperCos1 Vector Kit according to manufacturer’s instruction (Stratagene). The library was packaged using phage extracts and transduced into the *E. coli* LE392. About 2600 resulting transductants were picked up and transferred to twenty-seven 96-well microtiter plates containing 150 μL LB medium supplemented with Kan (50 μg/mL). After overnight incubation at 37 °C, 30 μL *E. coli* broth in every microtiter pore was absorbed and mixed every 12 clones in a horizontal line and every 8 clones in a vertical line for each 96-well plate. Glycerol was added to the remaining broth of the clones (20% final concentration) for permanent stock. The DNA of mixed clones was extracted as templates for PCR screening.

The primer pairs targeted the cytochrome P450 oxidase gene (NcmG-SF and NcmG-SR), Dieckmann cyclase gene (NcmC-SF and NcmC-SR) and DH domain at NcmAII gene (DH-SF and DH-SR) were designed and they were used as PCR primers to screen *S. syringae* NRRL B-16468 genomic library (Table 2). The positive clones were selected from the genomic library. The cosmids were extracted and further analyzed by terminal-sequencing. The PCR reaction (20 mL volume) contained 2 μL 10 × PCR buffer, 1.6 μL dNTPs (2.5 mM), 0.4 μL forward primer (10 μM), 0.4 μL reverse primer (10 μM), 1 μL dimethylsulfoxide (DMSO), 1 μL DNA template, 0.1 μL rTaq (5 U/μL), and 13.5 μL ddH_2_O. The following PCR program was used: 94 °C, 4 min, 30 cycles of 94 °C, 45 s, 59 °C, 45 s, 72 °C, 1 min, and a final extension cycle at 72 °C, 10 min. Eventually, two cosmids p5-C-9 and p2-H-12 were chosen for further gene-inactivation experiments.

**Table 2:** Primer pairs used in this study

Primers sequences (5′–3′)	
NcmG-delF	CTGCTGGGCGCCGACGTGCCGCGCACCACGCCGCGGGTGATTCCGGGGATCCGTCGACC
NcmG-delR	CAGGTCCGCCGCCGGTACCGCGAGCCGCAGGGTCGGGAATGTAGGCTGGAGCTGCTTC
NcmB-delF	CTGGCCTGCGCCGAACCGCCCGCGCCCGCCTCGCCCGTCATTCCGGGGATCCGTCGACC
NcmB-delR	GCCCCGGTGCCCCGCGGGCAGGCGGGCGCCGGGGCCCGCTGTAGGCTGGAGCTGCTTC
NcmL-delF	CGCAGCCTGGAGGTGTTCGACGACCTGGGCGTCGTCGACATTCCGGGGATCCGTCGACC
NcmL-delR	GAACCCGAAGAGCGTGAAGTGCGGGCCGCGCTGCGCGTCTGTAGGCTGGAGCTGCTTC
NcmG-tF	GAGGTCCGGCAGGTGCTGTC
NcmG-tR	GACGACCTTGGCGGTGTGCC
NcmB-tF	CGGGAGTACTGGCGGCAGC
NcmB-tR	GGTCCAGCAGGTCGGCCAGCA
NcmL-tF	CTGATCATCGACAAGGACTC
NcmL-tR	GGACGAGCACCAGCGCGTCC
DH-SF	GCTCGGTGTTCCTGGACCTGGC
DH-SR	GCAGTTCGAAGCCGCTCCACAG
NcmG-SF	GTCCACCGCGACGCCATAC
NcmG-SR	CGGCCAGGTAGTCCTTGAGCC
NcmC-SF	GGGCGGTGCTCGGGTTCT
NcmC-SR	GCAGGTCGGCGTGGTGGA

### Generation of *S. syringae* mutant strains

λ-RED recombination technology was employed to inactivate the target gene *ncmB, ncmL* and *ncmG* according to literature previously reported [14]. The primer pairs used for PCR-targeting are listed in Table 2. The fragment *oriT/acc(3)IV* cassette was used to replace partial gene region of *ncmB* or *ncmL* in p5-C-9 to generate cosmid pMoS1001 (*ΔncmB*) or pMoS1002 (*ΔncmL*). For *ncmG*, partial gene region was replaced by fragment *oriT/acc(3)IV* cassette in cosmid p2-H-12 and plasmid pMoS1003(*ΔncmG*) were generated. After verified by PCR and restriction enzyme digestion analysis, the correct mutated cosmids were introduced into *E. coli* ET12567/pUZ8002 and conjugated with wild type *S. syringae* spores. The wild type *S. syringae* spores were germinated in LB medium for 4–5 h at 30 °C, 200 rpm. The *E. coli* ET12567/pUZ8002 containing each mutated cosmid was grown in LB medium supplemented with Kan (50 μg/mL), Amp (100 μg/mL), Cml (25 μg/mL) and Apr (50 μg/mL) to OD_600_ = 0.6–0.8. Then the cells were harvested, washed twice with LB medium, mixed with germinated wild type spores and plated on ISP4 medium. The plates were incubated at 30 °C for 24 h. Then, each plate was covered by 800 μL sterile water supplemented with 30 μL TMP (50 mg/mL) and 30 μL Apr (50 mg/mL). The plates were continued incubated at 30 °C for 7–10 days until exconjugants appeared. Double cross-over mutants were first selected by the phenotype of Kan sensitive (Kan^S^) and Apr resistant (Apr^R^), and the genotype of the mutants were further confirmed by PCR. Finally, the mutant strains *S. syringae* MoS-1001 (*ΔncmB*), *S. syringae* MoS-1002 (*ΔncmL*) and *S. syringae* MoS-1003 (*ΔncmG*) were obtained.

### Fermentation and analysis of *S. syringae* and mutant strains

*Saccharothrix syringae* wild type and mutant strains were inoculated in 250 mL flasks with 50 mL medium and incubated on a rotary shaker at 28 °C, 200 rpm. After 7 days fermentation, each of the 50 mL culture was added with 100 mL ethyl acetate and then vigorously mixed for 30 min. The ethyl acetate phase was evaporated to dryness to yield a residue. The residue was dissolved in 1 mL methanol and centrifuged, then, the supernatant was subjected to HPLC analysis. Analytical HPLC was performed on Agilent 1260 HPLC system (Agilent Technologies Inc., USA) equipped with a binary pump and a diode array detector using a Phenomenex Prodigy ODS column (150 × 4.60 mm, 5 μ) with UV detection at 355 nm. The mobile phase comprises solvent A and B. Solvent A consists of 15% CH_3_CN in water supplemented with 0.1% trifluoroacetic acid (TFA). Solvent B consists of 85% CH_3_CN in water supplemented with 0.1% TFA. Samples were eluted with a linear gradient from 5 to 90% solvent B in 20 min, followed by 90–100% solvent B for 5 min, then eluted with 100% solvent B for 3 min, at a flow rate of 1 mL/min and UV detection at 355 nm.

### Isolation of new produced nocamycin derivatives from *ΔncmG* mutant strain

Two-step fermentation was used to culture *ΔncmG* mutant strain. 250 mL flask containing 50 mL medium was used as seed culture and 500 mL flask containing 100 mL medium was used as fermentation medium. Appropriate spores were inoculated to seed culture and grown at 28 °C, 200 rpm for 3 days. Then, 5 mL seed medium was inoculated to 100 mL fermentation medium and continued 7 days culture. 15 L liquid medium was used in total. After incubation, the culture broth was collected and centrifuged. The supernatant was extracted by ethyl acetate for three times and the mycelium was extracted by acetone for three times. Then, the entire organic phases were evaporated to dryness to yield crude extract. The crude extract was dissolved in a mixture of CH_3_OH: CHCl_3_ (1:1) and mixed with appropriate amount of silica gel (100–200 mesh, Qingdao Marine Chemical Corporation, China). The sample was applied on normal phase silica gel column chromatography and eluted with CHCl_3_-CH_3_OH (100:0–50:50) to give 10 fractions. All the fractions were analyzed by HPLC. Fraction 4 and 5 contained the major targeted compound nocamycin III and fractions 7 and 8 contained the major targeted compound nocamycin IV. The fractions 4–5 and fractions 7–8 were further purified on reverse phase C-18 silica gel (YMC, Japan) by using medium-pressure liquid chromatography (MPLC, Agela corporation, China) eluted by a linear gradient from 20 to 90% CH_3_CN in water, respectively. The sub-fractions contained targeted compounds were further purified by Sephadex LH-20 (GE healthcare, Sweden) gel filtration chromatography to afford the purified nocamycin III and nocamycin IV.

### Spectroscopy analysis of new produced nocamycin derivatives

^1^H and ^13^C NMR spectra of nocamycin III and nocamycin IV were recorded at 25 °C on Bruker AV 500 instruments. HR–ESI–MS spectra data were acquired on a Waters micro MS Q-Tof spectrometer.

## Results

### Sequencing and identification of nocamycin gene cluster

**Fig. 2:**
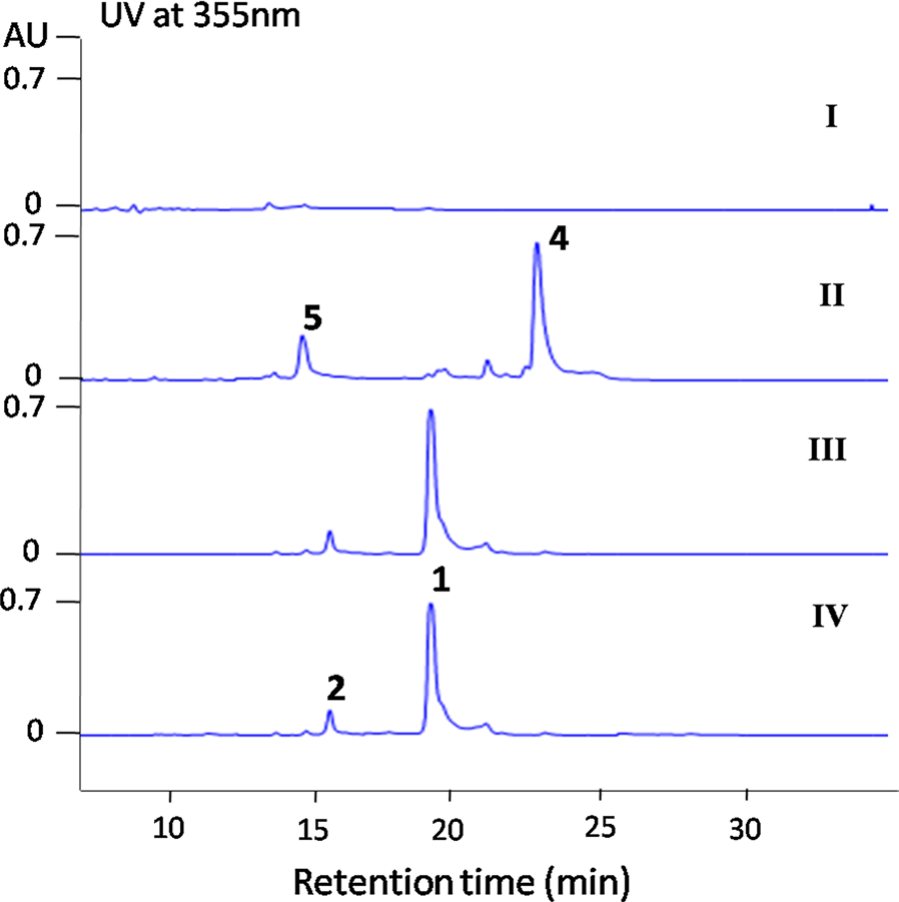
HPLC analysis of *S. syringae* and its mutant strain. I* ncmB* deletion mutant strain *S. syringae* MoS1001; II* ncmG* deletion mutant strain *S. syringae* MoS1003; III* ncmL* deletion mutant strain *S. syringae* MoS1002; IV* S. syringae* wild type strain. 1, 2, 4, 5 represent for nocamycin I, II, III and IV, respectively

**Fig. 3:**
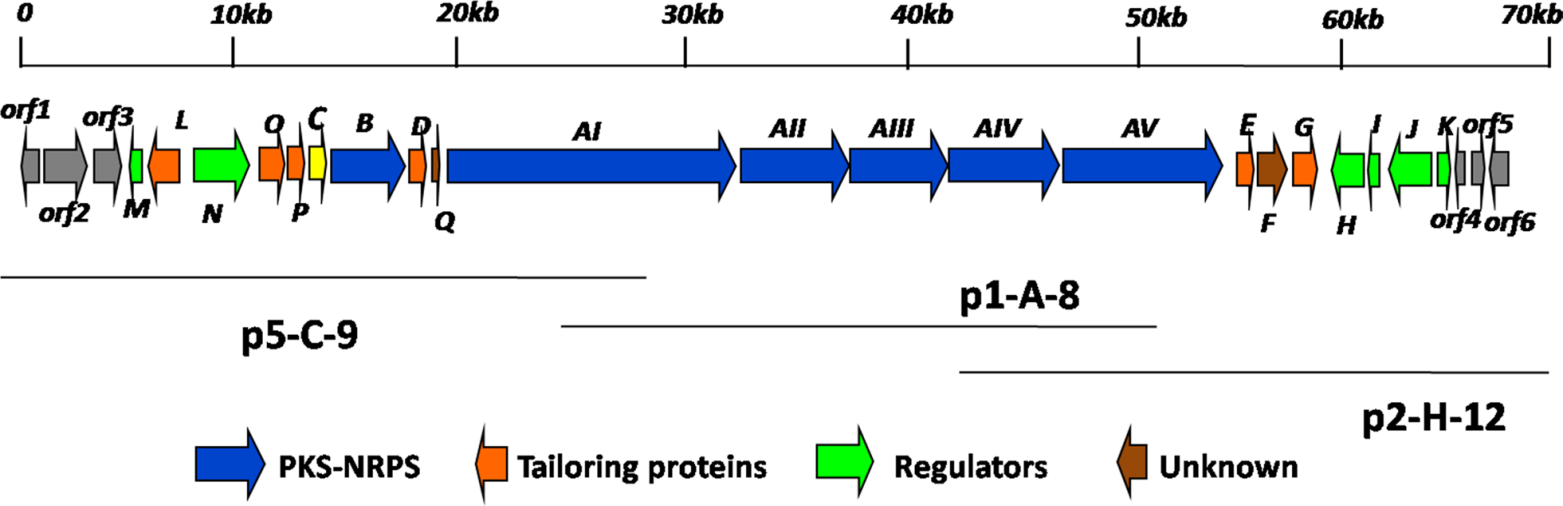
Gene organization of nocamycin biosynthetic gene cluster

*Saccharothrix syringae* NRRL B-16468 genome was shotgun sequenced by Hiseq4000 technologies and the sequence reads were assembled into 10.8 Mb nucleotides. Then, *S. syringae* NRRL B-16468 genome data was analyzed by using online antiSMASH tool [27]. AntiSMASH analysis results demonstrated that a hybrid PKS-NRPS gene cluster designated as *Ncm* seems to be the candidate responsible for nocamycin biosynthesis since it shows high similarity to tirandamycin biosynthetic gene cluster. In the *Ncm* gene cluster, some deduced gene products such as NcmC, NcmE, NcmF and NcmB show high similarity to TrdC, TrdE, TrdF and TrdB originated from tirandamycin biosynthetic pathway, respectively [14]. Thus, we assumed that this cluster is probably involved in nocamycin biosynthesis. We then screened *S. syringae* genomic library by using PCR method with the primer pairs targeted at *ncmG*, *ncmC* and dehydratase (DH) domain at module 4. In total of eight positive cosmids were obtained. The eight cosmids were end-sequenced and two cosmids p2-H-12 and p5-H-9 were used for further gene inactivation experiments. To verify our hypothesis, a gene *ncmB* encoding a NRPS was inactivated to afford the strain *S. syringae* MoS-1001 (Additional file 1: Figure S1). HPLC analysis of the extract of *S. syringae* MoS-1001 fermentation broth revealed that *S. syringae* MoS-1001 failed to produce nocamycin I and II (Fig. 2I) completely, indicating *ncmB*’s involvement in nocamycin biosynthesis. This result also demonstrated this PKS-NRPS gene cluster is responsible for nocamycin biosynthesis. On basis of bioinformatics analysis, about 61 kb DNA locus consisted of 21 open reading frames (ORFs) whose deduced products are likely to be involved in nocamycin biosynthesis (Fig. 3; Table 3). Corresponding homologues and deduced function of each *ncm* gene are listed in Table 3. The sequence data of nocamycin biosynthesis in this study have been deposited in Genbank under accession number KY287782.

**Table 3:** Deduced functions of genes in the nocamycin biosynthetic gene cluster

Gene	Length (amino acids)	Closest similar protein accession number, origin, identity/similarity (%)	Deduced function
*Orf1*	306	KOX27604, *Saccharothrix* sp. NRRL B-16348	Short-chain dehydrogenase
*Ofr2*	664	KOX27605.1, *Saccharothrix* sp. NRRL B-16348, 81/86%	Helicase
*Orf3*	455	WP_053719771, *Saccharothrix* sp. NRRL B-16348	Hypothetical protein
*NcmM*	225	SCD95979, *Streptomyces* sp. PalvLS-984, 68/79%	Transcriptional regulator
*NcmL*	511	EJI98707, *Rhodococcus* sp. JVH1, 50/60%	Monooxygenase
*NcmN*	911	AFI57028 (QmnRg4), *Amycolatopsis orientalis*, 43/55%	LuxR family regulator
*NcmO*	412	AFI57027 (QmnO), *Amycolatopsis orientalis*, 48/63%	Cytochrome P450 oxidase
*NcmP*	287	ACN29714 (NokK), *Nonomuraea longicatena*, 44/55%	Carboxylate *O*-methyltransferase
*NcmC*	272	ADY38535 (TrdC), *Streptomyces* sp. SCSIO1666, 45/58%	Dieckmann cyclase
*NcmB*	1119	ADY38536 (TrdD), *Streptomyces* sp. SCSIO1666, 56/66%	Non-ribosomal peptide synthetase
*NcmD*	271	AII10529, *Rhodococcus opacus*, 45/60%	Short-chain dehydrogenase
*NcmQ*	120	EJY55702, *Alicyclobacillus hesperidum* URH17-3-68, 41/55%	Glyoxalase/bleomycin resistance protein
*NcmAI*	4915	CBA11584 (SlgA1), *Streptomyces lydicus*, 54/64%	Type I polyketide synthase
*NcmAII*	1786	EHY88978, *Saccharomonospora azurea* NA-128, 54/65%	Type I polyketide synthase
*NcmAIII*	1554	CCF23202, *Streptomyces hygroscopicus*, 60/69%	Type I polyketide synthase
*NcmAIV*	1786	CCF23202.1, *Streptomyces hygroscopicus*, 56/66%	Type I polyketide synthase
*NcmAV*	2679	AEP40935.1, *Nocardiopsis* sp. FU40, 50/60%	Type I polyketide synthase
*NcmE*	273	ADC79643 (TamE), *Streptomyces* sp. 307-9, 60/76%	Glycoside hydrolase
*NcmF*	487	ADY38538 (TrdF), *Streptomyces* sp. SCSIO1666, 50/61%	Prenyltransferase
*NcmG*	397	ADZ45320(Mur7), *Streptomyces* sp. NRRL 30471, 51/64%	Cytochrome P450 oxidase
*NcmH*	543	CAH10178 (ChaT1), *Streptomyces chartreusis*, 42/60%	Multiple drug transporter
*NcmI*	197	KKZ83567, Rhizobium phaseoli Ch24-10, 41/61%	PadR family transcriptional regulator
*NcmJ*	713	WP_037345636, *Sciscionella* sp. SE31, 69/79%	AAA family ATPase
*NcmK*	213	ADY38543(TrdK) *Streptomyces* sp. SCSIO1666, 49/64%	TetR family transcriptional regulator
*Orf4*	110	GAT66653, Planomonospora sphaerica, 43/54%	Ohr subfamily peroxiredoxin
*Orf5*	232	GAT10151, *Mycobacterium novocastrense*, 64/73%	Ubiquinone biosynthesis methyltransferase UbiE
*Orf6*	322	KDO05396, Amycolatopsis mediterranei, 68/77%	(2Fe–2S) ferredoxin

### Linear chain assembly and releasing

**Fig. 4:**
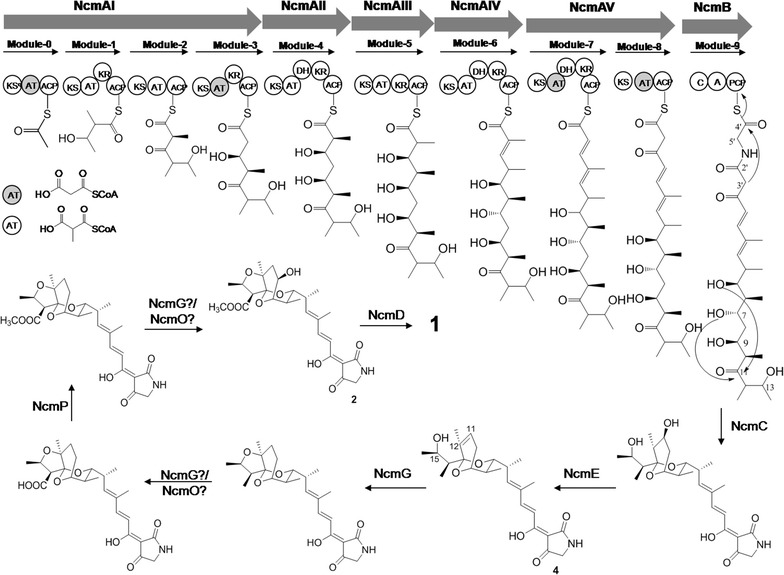
The putative biosynthetic pathway of nocamycins

Hybrid PKS-NRPS are employed to construct the backbone structure of nocamycin. Five type I PKS genes *ncmAI*, *ncmAII*, *ncmAIII*, *ncmAIV* and *ncmAV* transcribed in the same direction were identified in the gene cluster (Fig. 3). The deduced products of the five PKS genes were constituted by four, one, one, one and two modules respectively to assemble the polyketide backbone (Fig. 4). Each PKS module minimally contains ketosynthase (KS), acyltransferase (AT) and acyl carrier protein (ACP) domains. The conserved motifs from PKS modules in nocamycin gene cluster are listed in Additional file 1: Table S1. Except for loading module, M2 and M8, each module possess a ketoreductase (KR) domain with conserved active motif. KR domain in module M5 is the only KR domain contains the characteristic of A-type KRs, and all the other KR domains display the conserved motif characteristic for the B-type KRs [28]. A characteristic KS^Q^ domain of loading module indicated that a malonoyl–CoA might be used to provide acetate as starter unit, and this phenomenon was observed in tirandamycin and streptolydigin gene clusters [10, 14]. As shown in Table 4 and Fig. 4, the AT domains in extension modules M3, M7 and M8 display conserved active motif specific for malonate-CoA incorporation [29, 30], whereas AT domains in extension modules M1, M2, M4, M5 and M6 show conserved active motif specific for methylmalonate-CoA incorporation [29, 30], which is in good agreement with the polyketide carbon skeleton. There are three DH domains with conserved active motif HXXXGXXXXP distributed in module M4, M6 and M7 [31].

**Table 4:** ^1^H and ^13^CNMR spectroscopic data for nocamycin III (**4**) and nocamycin IV (**5**)

Position	**4** ^**a**^		**5** ^**b**^	
	*δ*_C_ type	*δ*_H_ mult. (*J* in Hz)	*δ*_C_ type	*δ*_H_ mult. (*J* in Hz)
1	175.5		Not observed	
2	116.6	7.17, d (15.7)	Not observed	7.33, d (14.2)
3	150.3	7.60, d (15.7)	Not observed	7.56, d (15.6)
4	135.3		136.5	
5	144.5	6.05, d (10.2)	144.0	6.04, d (9.4)
6	34.6	2.89, m	35.7	2.97, m
7	78.2	3.66, dd (11.2, 2.0)	79.3	3.75, dd (11.3, 1.6)
8	35.2	2.0, m	36.7	1.95, m
9	70.7	4.0, t (6.3)	71.7	4.04, t (6.2)
10	23.9	1.98, m; 2.4, m	24.4	2.16, m; 2.43, m
11	125.9	5.84, d (3.5)	125.8	6.23, d (4.2)
12	130.8		135.7	
13	101.3		100.8	
14	43.7	1.94, m	45.2	1.89, dd (14.1, 7.0)
15	68.9	4.29, dq (8.5, 6.3)	69.0	4.36, dq (12.5, 6.3)
16	20.7	1.23, d (6.3)	20.6	1.22, d (6.3)
17	12.4	1.92, s	12.6	1.94, s
18	17.0	1.07, d (7.0)	17.7	1.09, d (6.8)
19	13.2	0.71, d (6.9)	13.4	0.79, d (6.9)
20	18.0	1.62, s	62.2	3.96, d (13.1); 4.07, d (14.9)
21	11.8	0.79, d (7.0)	11.2	0.85, d (6.9)
1′				
2′	176.7		Not observed	
3′	Not observed		Not observed	
4′	192.8		Not observed	
5′	51.7	3.84, s	52.0	3.79, s

^a^Measured in CDCl_3_

^b^Measured in MeOD

NcmB, a NRPS, shows most similarity to TrdD (56% identity/66% similarity) from *Streptomyces* sp. SCSIO1666 involved in tirandamycin biosynthetic pathway [14]. Three domains condensation (C), adenylation (A), and peptidyl carrier protein (PCP) are found in NcmD. The amino acid binding pocket DILQLGVI located in A domain is predicted to activate glycine, which is accord to nocamycin structure.

NcmC shows most similarity to TrdC (45% identity/58% similarity) from *Streptomyces* sp. SCSIO1666 involved in tirandamycin biosynthetic pathway [14]. TrdC and its analogues SlgC, KirHI have been determined as Dieckmann cyclases, and they catalyze the formation of tetramic acid or pyridone moiety [21]. Bioinformatics analyses revealed that NcmC also possesses the characteristic catalytic traid Cys-Asp-His (Additional file 1: Figure S4). Thus, in nocamycin biosynthesis pathway, NcmC is proposed to be responsible for the PK-NRP chain releasing and catalyze the formation of tetramic acid moiety.

### Genes involved in post-tailoring steps

After linear chain released from PKS-NRPS and formation of tetramic acid moiety, several post tailoring processes including oxolane ring system, C-10 hydroxyl/ketone group, C-14 methoxycarbonyl group are required to synthesis nocamycin I. Within the identified gene cluster, there are six genes encoding two cytochrome P450 monooxygenase (*ncmO* and *ncmG*), one monooxygenase (*ncmL*), one carboxylate O-methyltransferase (*ncmP*), one short chain dehydrogenase (*ncmD*) and one glycoside hydrolase (*ncmE*) are likely to be involved in these steps.

The glycoside hydrolase NcmE shows identity to TrdE (60% identity/76% similarity) involved in tirandamycin biosynthesis [14]. In tirandamycin pathway, TrdE functions as a dehydratase and it is responsible for the formation of C11–C12 double bond [17]. Thus, we propose that NcmE is a dehydratase and it catalyzes the formation of C11–C12 double bond.

Both cytochrome P450 monooxygenases NcmG and NcmO possess the highly conserved heme-binding domain (GXXXCXG), K-helix (EEXLL) and oxygen binding region (Additional file 1: Figure S5) [32, 33]. NcmG shows similarity to Mur7 (51% identity/64% similarity) involved in muraymycin biosynthesis [34]. NcmO shows similarity to QnmO (48% identity/63% similarity) involved in quartromicin biosynthesis [35]. Since at least three oxidative tailoring steps, including the formation of tetrahydrofuran fused in bicyclic ketal structure, C-10 hydroxyl and C-14 carboxyl group are required, NcmG or NcmO is proposed to be bifunctional. Sequence alignments analysis revealed that NcmO and NcmG are distinct from the cytochrome P450 oxidases TrdI/TamI, SlgO1 and SlgO2 involved in tirandamycin or streptolydigin biosynthetic pathway [10, 15] (Additional file 1: Figure S6). The reason for this phenomenon may attribute to the different oxidative modification in bicyclic ketal structure of nocamycin, streptolydigin and tirandamycin.

Within nocamycin gene cluster, only *ncmP* encodes for a SAM-dependent carboxylate *O*-methyltransferase and it shows identity to NokK (44% identity/55% similarity) and NivG (43% identity/53% similarity). Both NokK and NivG are proposed to catalyze methyl esterification of the carboxylate group in biosynthesis of K-252a and nivetetracyclates, respectively [36, 37]. Hence, it should be evident that NcmP serves as the best candidate responsible for methyl esterification during nocamycin biosynthetic pathway.

NcmL shows similarities to monooxygenase from a series of actinomycetes. BLAST analysis revealed that NcmL displays FAD-binding domain (pfam01494). Unlike bicovalent flavinylation protein TrdL/TamL involved in tirandamycin biosynthetic pathway, NcmL has no conserved His and Cys dual active site residues that distributed in 8α-histidyl and 6-S-cysteinyl FAD linked monooxygenase family (Additional file 1: Figure S7) [15, 16]. To investigate the function of NcmL in nocamycin biosynthesis, the gene *ncmL* was inactivated (Additional file 1: Figure S2). The fermentation broth of *ΔncmL* mutant strain was analyzed by HPLC (Fig. 2III). The results revealed that the titer of nocamycin I and nocamycin II in *ΔncmL* deletion strain is identical to that in wild type, indicating NcmL is not involved in nocamycin biosynthesis.

The putative product of *ncmD* shows identity to a series of short chain dehydrogenase (SDR) family oxidoreductase originated from various bacteria. NcmD shares the Rossmann fold NAD-binding motif and characteristic NAD-binding and catalytic sequence patterns [38]. NcmD shows closet similarity to BatM (40% identity/56% similarity) which was proposed to catalyze the conversion from hydroxyl to ketone in C-17 position during kalimantacin/batumin-related polyketide antibiotic biosynthesis [39]. Thus, NcmD is proposed to be the candidate to convert hydroxy to ketone in C-10 position.

### Genes involved in regulation, resistance and unknown functions

Five genes related to regulation and resistance are easily discerned from the nocamycin biosynthetic gene cluster. *NcmN* encodes for a LuxR family regulator and it shows similarity to a series of regulators from different actinomycetes, including QmnRg4 (43% identity/55% similarity) from *Amycolatopsis orientalis* involved in quartromicin biosynthesis and TamH (39% identity/52% similarity) involved in tirandamycin biosynthesis [14, 35]. The characteristic C-terminal helix-turn-helix (HTH) DNA binding domain signature and a N-terminal ATP-binding domain represented by discernible Walker A (GxxGxGK) and Walker B (R/K-X(7-8)-H(4)-D) motifs present in all members of this family of regulatory proteins are found in NcmN [40]. NcmJ is similar to AAA family ATPase from different actinomycetes. AAA family ATPases are present in all kingdoms and they are often involved in DNA replication, repair, recombination and transcription [41]. NcmJ contains the Walker A and Walker B motifs, which is the hallmark of ATP-binding domain in these proteins [41]. NcmI encodes a PadR family transcriptional regulator and it shows similarities to several PadR-like proteins of unknown function from different actinomycetes. PadR-like proteins is a quite recently identified family of regulatory proteins, named after the phenolic acid decarboxylation repressor of *Bacillus subtilis* [42, 43]. The hallmark of this family transcriptional regulator is a highly conserved N-terminal winged helix-turn-helix (HTH) domain with about 80–90 residues [44, 45], which is also found in NcmI. NcmK encodes for a TetR family transcriptional regulator and it shows identity to TrdK (49% identity/64% similarity) involved in tirandamycin biosynthesis [14]. The characteristic N-terminal helix-turn-helix (HTH) DNA binding domain signature (pfam00440) presented in all members of this family of regulatory proteins has been found in NcmK.

NcmH, a major facilitator superfamily (MFS) transporter, shows identity to ChaT1 (42% identity/60% similarity) from *Streptomyces chartreusis* involved in antitumor agent chartreuse in biosynthesis pathway, is a candidate protein for resistance [46]. NcmQ is similar to the proteins belong to glyoxalase/bleomycin resistance protein/dioxygenase superfamily. The exact role of NcmQ in nocamycin biosynthesis is unclear and we assume that NcmQ is likely involved in resistance.

The deduced product of *ncmF* shows similarity to a series of prenyltransferase, including TrdF (50% identity/61% similarity) involved in tirandamycin biosynthesis and SlgF (51% identity/60% similarity) involved in streptolydigin biosynthesis, respectively [10, 14]. Previous studies on TrdF and SlgF demonstrated that both the proteins show no relationship with tirandamycin or streptolydigin biosynthesis [10, 14]. Thus, we hypothesize that NcmF maybe not involved in nocamycin biosynthesis.

### Inactivation of *ncmG* and isolation the new derivatives from the mutant strain

Cytochrome P450 oxidases are often play important roles in post-tailoring steps during antibiotic biosynthesis. Generally, oxygenation modification is a vital approach to improve bioactivity of parent molecule. To obtain more nocamycin derivatives, we inactivated *ncmG* by λ-RED/ET technology and generated *ΔncmG* mutant strain *S. syringae* MoS1003 (Additional file 1: Figure S3). HPLC analysis revealed that *S. syringae* MoS1003 abolished nocamycin I and nocamycin II production completely and two new peaks with similar characteristic UV absorption to these of nocamycin I and nocamycin II are detected (Fig. 2II). Then, A 15-L scale fermentation of *ΔncmG* mutant strain led the purification of nocamycin III and nocamycin IV. The structures of nocamycin III and nocamycin IV were determined by multiple spectroscopy data analyses. Both nocamycin III and IV are new nocamycin derivatives. Compared to nocamyin I and II, nocamycin III and IV show less oxidative modification, lacking of tetrahydrofuran ring, C-10 and C-21 modification.

**Fig. 5:**
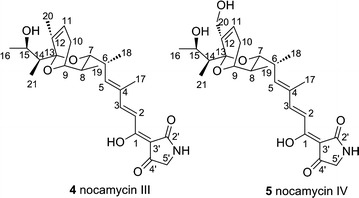
Structure of nocamycin III and IV

The molecular formula of nocamycin III (**4**) is C_25_H_35_NO_6_ (*m/z* = 445.25), which was determined by HR–ESI–MS ([M − H]^−^ *m/z* = 444.2422, [M + H]^+^ *m/z* = 446.2548, [M + Na]^+^ *m/z* = 468.2354) (Additional file 1: Figure S8). Comparisons of the ^1^H and ^13^C NMR spectroscopic data of nocamycin III to those of nocamycin I (Bu-2313B) suggested that they share a similar structure. Complete spectral data including COSY, HSQC, and HMBC spectra were also acquired (Additional file 1: Figures S10–S16), thereby allowing full assignments of the ^1^H and ^13^C signals (Table 4). Comparisons of the ^1^H and ^13^C NMR data for nocamycin I and nocamycin III revealed that the tetrahydrofuran ring is not closed and a Δ^11,12^ double bond is apparent in nocamycin III. HMBC correlations from H-20 to C-11, C-12, and C-13, and the COSY correlations of H-10/H-11 further substantiated these assignments. Additionally, H-15 was shifted to δ_H_ 4.29 due to the ring opening, relative to the same position of the cyclic form. A keto group in nocamycin I was replaced by a methylene group (δ_H_, 1.98, 2.4; δ_C_ 23.9) at C-10 in **4**, which was confirmed by the HMBC correlations from H-8, H-9, and H-11 to C-10 and from the COSY correlations of H-9/H-10*α*, and of H-10*β*/H-11. Another obvious difference observed from the ^1^H and ^13^C spectroscopic data was the absence of a –COOCH_3_ in **4** compared to that of nocamycin I. In turn, a methyl group (δ_H_, 0.79, δ_C_ 11.8) was found to be attached at C-14. Cross peak of H-14/H-21 in the COSY spectrum and the HMBC correlations from H-21 to C-13, C-14 and C-15 further confirmed this assignment. Inspection of other NMR data for nocamycin III revealed other structural elements are identical to those of nocamycin I. Consequently, the structure of nocamycin III was elucidated as shown in Fig. 5.

Nocamycin IV (**5**) was isolated as a yellowish amorphous solid. Its molecular formula was determined as C_25_H_35_NO_7_ (*m/z* = 461.24) by HR–ESI–MS ([M − H]^−^ *m/z* = 460.2355, [M + H]^+^ *m/z* = 462.2503, [M + Na]^+^ *m/z* = 484.2303) (Additional file 1: Figure S9), 16 mass units greater than that of nocamycin III, indicating one more oxygen atom than that of nocamycin III. Complete spectral data including COSY, HSQC, and HMBC spectra were also acquired (Additional file 1: Figures S17–S21), thereby allowing full assignments of the ^1^H and ^13^C signals (Table 4). It shared a similar structure to that of nocamycin III, except that a methyl signal at δ_H_ 1.62 was disappeared and an oxygen-bearing methylene signal at δ_H_ 3.96 and 4.07 occurred. Key HMBC correlations from H-20 to C-12 and from H-11 to C-12 further confirmed the location of the –CH_2_OH group at C-12. Thus, the structure of nocamycin IV was elucidated as 20-hydroxy-nocamycin III (Fig. 5).

## Discussion

In this study, the gene cluster responsible for nocamycin biosynthesis identified from *S. syringae* consists of 21 ORFs: 12 coding for structural proteins, seven involved in regulator and resistance and two with unknown function. Like the reported biosynthetic gene clusters of tirandamycin and streptolydigin, a hybrid PKS-NRPS mechanism is employed to assemble the chain PK-NPR backbone by co-linearity rule [10, 13, 14]. The core structure of nocamycin is bicyclic ketal unit and tetramic acid moiety. To date, tetramic acid structure has been identified in numerous natural products and four phylogenetically different family enzymes have been characterized to catalyze the tetramic acid formation through Dieckmann cyclisation reaction [21, 47–50]. In previous report, TrdC and its homologous protein SlgL have been characterized as Dieckmann cyclases to catalyze the formation of tetramic acid moiety in tirandamycin and streptolydigin biosynthetic pathway, respectively [21]. Thus, it is plausible to assume that NcmC, the homologous protein to TrdC, is employed to generate tetramic acid moiety through Dieckmann cyclisation during nocamycins biosynthetic pathway [21].

Formation of bicyclic ketal ring represents the most intriguing issue of nocamycin family natural products, which is not fully understood. In our previous study, an abnormal DH at module 3 in tirandamycin PKS was proposed to be involved in spiroketal structure formation [14]. Comparing with tirandamycin and streptolydigin gene clusters, it is important to notice that all the three gene clusters possess a similar unexpected DH domain with conserved active motif in the corresponding PKS. This abnormal DH domain at module 4 are likely not to catalyze the dehydration reaction to afford C-10 and C-11 double bond because the C-11 hydroxy group is absolutely required for the C-13 spiroketal group formation and no nocamycin derivatives possess C-10 and C-11 double bond have been identified. Recently, linear 7,13,9,13-diseco-tirandamycin derivative tirandamycin K, a shunt pathway product in tirandamycin pathway, was isolated from *Streptomyces* sp. 307-9 and its P450 monooxygenase disruption mutant strain [51]. C-9 hydroxyl in tirandamycin K clearly indicates that DH3-catalyzed dehydration can be avoided, and it also provides evidence to support the mechanism that DH3 is involved in bicyclic ketal formation [51]. Due to the high similarity in polyketide structure and domains organization of PKS between tirandamycin, nocamycin and streptolydigin gene clusters, the abnormal DH catalytic mechanisms are likely to be common spiroketalization mechanisms in these three pathways.

Based on bioinformatics and genetic engineering data, post tailoring steps of nocamycin can be predicted as follows (Fig. 4). Firstly, the earliest intermediate released from the PCP protein possesses a hydroxyl group in C-11 position, NcmE catalyze the dehydration process to afford nocamycin III. Next, nocamycin III undergoes several oxidative and one methyl esterification steps to produce nocamycin I. At last, NcmD catalyzes the dehydrogenation process to afford nocamycin II. Comparisons of gene clusters of tirandamycin and nocamycin revealed an interesting phenomenon that the post-tailoring enzymes involved in modification of similar structure are varied. In tirandamycin biosynthetic pathway, a FAD-dependent dehydrogenase TrdL/TamL is responsible for the conversion from C-10 hydroxyl to C-10 ketone [15, 16]. In our initial hypothesis, a TrdL/TamL homologous protein is predicted to be responsible for the same process, however, no TrdL/TamL homologous protein has been observed within the gene cluster. Although NcmL shows FAD-binding domain, it lacks the conserved bicovalent FAD linked active sites to that in TamL/TrdL [15, 16]. Meanwhile, the gene inactivation experiments revealed that NcmL shows no relationship to nocamycin biosynthesis, and this result also indicates that diversified modification mechanism occurred in this class of natural products. Overview the gene cluster, the short-chain dehydrogenase NcmD is the best candidate to catalyze the last C-10 dehydrogenation step in nocamycin biosynthetic pathway. The complex oxidative modifications including formation of fused oxolane ring system in bicyclic ketal moiety and the conversion from methyl group to carboxyl are intriguing issues, and the two cytochrome P450 oxidase NcmG and NcmO are expected to be involved in these steps. Two new derivatives nocamycin III and nocamycin IV lacking of closed tetrahydrofuran ring from *ΔncmG* mutant strain indicates NcmG’s involvement in the formation of the fused oxolane ring system. In terms of oxolane ring system formation, four different biosynthetic routes have been envisioned [52–55]. The mechanism of tetrahydrofuran ring in nocamycin is proposed to be similar to that in nonactin biosynthesis pathway [52]. NcmG is likely to catalyze conjugate addition of C-15 hydroxyl groups to the adjacent C-11 and C-12 alkenyl moiety to form oxolane ring (Fig. 4). We notice that the C-20 hydroxyl in nocamycin IV is similar to C-18 hydroxyl in tirandamycin B. In tirandamycin biosynthetic pathway, a multifunctional cytochrome P450 TamI has been verified to be responsible for the formation of C-18 hydroxyl group [15]. However, C-20 hydroxylation modification is not required in nocamycin biosynthetic pathway (Fig. 4). Thus, we hypothesize that nocamycin IV is probably a shunt product in nocamycins biosynthetic pathway and an oxidase located elsewhere of the genome can catalyze the hydroxylation process. Considerations of several oxidative modifications are required to afford nocamycin II, one of NcmG and NcmO is potentially responsible for more than one oxidative tailoring steps. Elucidation of the exact roles of NcmG and NcmO and the timing of modification in nocamycin biosynthesis is our ongoing project.

Up to now, the biosynthetic gene clusters responsible for streptolydigin, tirandamycin and nocamycin biosynthesis have been identified. Comparisons of the three gene clusters will help us deeply understand the biosynthetic mechanisms of this small class of natural products. The genetic insights and elucidations of enzyme function will facilitate us to rationally generate new derivatives with improved pharmacological property by manipulating biosynthetic pathway.

## Conclusion

The nocamycin I and II, bearing a tricyclic ketal moiety, belong to acyl tetramic acid natural products and they display broad antimicrobial activity. In this report, we identify nocamycins biosynthetic gene cluster from rare actinomycete *Saccharothrix syringae,* which provides us the genetic insights into nocamycins biosynthesis and enzyme candidates for several intriguing biochemical transformations. Inactivation of cytochrome P450 monoxygenase NcmG led to isolation of two novel nocamycin derivatives from the mutant strain. Based on gene cluster data and new derivatives isolated from gene inactivation mutant strain, a putative biosynthetic pathway of nocamycin is proposed. These findings provide insights into further investigation of nocamycin biosynthetic mechanism, and also set the stage to rationally engineer new nocamycin derivatives via manipulating biosynthetic pathway.

## Additional file

**Additional file 1: Table S1.** Conserved motifs from PKS modules in nocamycin gene cluster. **Figure S1.** Inactivation of *ncmB* by gene disruption. **Figure S2.** Inactivation of *NcmL* by gene disruption. **Figure S3.** Inactivation of *NcmG* by gene disruption. **Figure S4.** Multiple sequence alignment of NcmC and its homologous protein sequence. **Figure S5.** Multiple sequence alignments of the cytochrome P450 domains of NcmG and NcmO. **Figure S6.** Unrooted phylogenetical tree of NcmO and NcmG with TrdI, SlgO1, SlgO2 and other cytochrome P450s. **Figure S7.** Multiple sequence alignments of NcmL with confirmed proteins that contain biocovently linked FAD cofactor. **Figure S8.** HR–ESI–MS of nocamycin III (**4**). **Figure S9.** HR–ESI–MS of nocamycin IV (**5**). **Figure S10.** 1H NMR (700 MHz) spectrum of compound 4 in CDCl3.** Figure S11.** 13C NMR (176 MHz) spectrum of compound **4** in CDCl3. **Figure S12.** DEPT 135 spectrum of compound **4** in CDCl_3_. **Figure S13.** 1H–1H COSY spectrum of compound 4 in CDCl_3_. **Figure S14.** HSQC spectrum of compound 4 in CDCl3. **Figure S15.** HMBC spectrum of compound 4 in CDCl3. **Figure S16.** NOESY spectrum of compound 4 in CDCl3. **Figure S17.** 1H NMR (500 MHz) spectrum of compound 5 in MeOD. **Figure S18.** 13C NMR (125 MHz) spectrum of compound 5 in MeOD. **Figure S19.** 1H–1H COSY spectrum of compound 5 in MeOD. **Figure S20.** HSQC spectrum of compound 5 in MeOD. **Figure S21.** HMBC spectrum of compound 5 in MeOD.

## Abbreviations

ORF: open reading frame
NRPS: nonribosomal peptide synthetases
PKS: polyketide synthases
A domain: adenylation domain
PCP: peptidyl carrier protein
C domain: condensation domain
KS: ketosyntheatase
AT: acyltransferase
ACP: acyl carrier protein
DH: dehydratase
KR: ketoreductase
Apr^R^: apramycin resistant phenotype
Kan^S^: kanamycin sensitive phenotype
HPLC: high-performance liquid chromatography
HTH: helix-turn-helix
M: module
MS: mass spectrometry
NMR: nuclear magnetic resonance
TFA: trifluoroacetic acid
Amp: ampicillin
Apr: apramycin
Kan: kanamycin
Cml: chloroamphenicol
TMP: trimethoprim

## References

[1] Gauze G, Sveshnikova M, Ukholina R, Komarova G, Bazhanov V (1977). Formation of a new antibiotic, nocamycin, by a culture of *Nocardiopsis syringae* sp. nov. Antibiotiki..

[2] Brazhnikova M, Konstantinova N, Potapova N, Tolstykh I (1977). Physicochmemical characteristics of the new antineoplastic antibiotic, nocamycin. Antibiotiki..

[3] Horváth G, Brazhnikova MG, Konstantinova NV, Tolstykh IV, Potapova NP (1979). The structure of nocamycin, a new antitumor antibiotic. J Antibiot (Tokyo)..

[4] Tsunakawa M, Toda S, Okita T, Hanada M, Nakagawa S, Tsukiura H, Naito T, Kawaguchi H (1980). Bu-2313, a new antibiotic complex active against anaerobes II. Structure determination of Bu-2313 A and B. J Antibiot.

[5] Tsukiura H, Tomita K, Hanada M, Kobaru S, Tsunakawa M, Fujisawa M, Fujisawa K, Kawaguchi H (1980). Bu-2313, a new antibiotic complex active against anaerobes I. production, isolation and properties of Bu-2313 A and B. J Antibiot.

[6] Bansal M, Dhawan V, Thadepalli H (1982). In vitro activity of Bu-2313B against anaerobic bacteria. Chemotharapy..

[7] Toda S, Nakagawa S, Naito T (1980). Bu-2313, a new antibiotic complex active against anaerobes III. Semi-synthesis of Bu-2313 A and B. J Antibiot.

[8] Tuske S, Sarafianos SG, Wang X, Hudson B, Sineva E, Mukhopadhyay J, Birktoft JJ, Leroy O, Ismail S, Clark AD, Dharia C, Napoli A, Laptenko O, Lee J, Borukhov S, Ebright RH, Arnold E (2005). Inhibition of bacterial RNA polymerase by streptolydigin: stabilization of a straight-bridge-helix active-center conformation. Cell.

[9] Temiakov D, Zenkin N, Vassylyeva MN, Perederina A, Tahirov TH, Kashkina E, Savkina M, Zorov S, Nikiforov V, Igarashi N, Matsugaki N, Wakatsuki S, Severinov K, Vassylyev DG (2005). Structural basis of transcription inhibition by antibiotic streptolydigin. Mol Cell.

[10] Olano C, Gómez C, Pérez M, Palomino M, Pineda-Lucena A, Carbajo RJ, Braña AF, Méndez C, Salas JA (2009). Deciphering biosynthesis of the RNA polymerase inhibitor streptolydigin and generation of glycosylated derivatives. Chem Biol.

[11] Carlson JC, Li S, Burr DA, Sherman DH (2009). Isolation and characterization of tirandamycins from a marine-derived *Streptomyces* sp. J Nat Prod.

[12] Horna DH, Gómez C, Olano C, Palomino-Schätzlein M, Pineda-Lucena A, Carbajo RJ, Braña AF, Méndez C, Salas JA (2011). Biosynthesis of the RNA polymerase inhibitor streptolydigin in *Streptomyces lydicus*: tailoring modification of 3-methyl-aspartate. J Bacteriol.

[13] Carlson JC, Fortman JL, Anzai Y, Li S, Burr DA, Sherman DH (2010). Identification of the tirandamycin biosynthetic gene cluster from *Streptomyces* sp. 307-9. ChemBioChem.

[14] Mo X, Wang Z, Wang B, Ma J, Huang H, Tian X, Zhang S, Zhang C, Ju J (2011). Cloning and characterization of the biosynthetic gene cluster of the bacterial RNA polymerase inhibitor tirandamycin from marine-derived *Streptomyces* sp. SCSIO1666. Biochem Biophys Res Commun.

[15] Carlson JC, Li S, Gunatilleke SS, Anzai Y, Burr DA, Podust LM, Sherman DH (2011). Tirandamycin biosynthesis is mediated by co-dependent oxidative enzymes. Nat Chem..

[16] Mo X, Huang H, Ma J, Wang Z, Wang B, Zhang S, Zhang C, Ju J (2011). Characterization of TrdL as a 10-hydroxy dehydrogenase and generation of new analogues from a tirandamycin biosynthetic pathway. Org Lett.

[17] Mo X, Ma J, Huang H, Wang B, Song Y, Zhang S, Zhang C, Ju J (2002). Δ(11,12) double bond formation in tirandamycin biosynthesis is atypically catalyzed by TrdE, a glycoside hydrolase family enzyme. J Am Chem Soc.

[18] Gómez C, Horna DH, Olano C, Palomino-Schätzlein M, Pineda-Lucena A, Carbajo RJ, Braña AF, Méndez C, Salas JA (2011). Amino acid precursor supply in the biosynthesis of the RNA polymerase inhibitor streptolydigin by *Streptomyces lydicus*. J Bacteriol.

[19] Gómez C, Olano C, Méndez C, Salas JA (2012). Three pathway-specific regulators control streptolydigin biosynthesis in *Streptomyces lydicus*. Microbiology.

[20] Gómez C, Horna DH, Olano C, Méndez C, Salas JA (2012). Participation of putative glycoside hydrolases SlgC1 and SlgC2 in the biosynthesis of streptolydigin in *Streptomyces lydicus*. Microb Biotechnol.

[21] Gui C, Li Q, Mo X, Qin X, Ma J, Ju J (2015). Discovery of a new family of Dieckmann cyclases essential to tetramic acid and pyridone-based natural products biosynthesis. Org Lett.

[22] Macneil DJ, Gewain KM, Ruby CL, Dezeny G, Gibbons PH, Macneil T (1992). Analysis of *Streptomyces-avermitilis* genes required for avermectin biosynthesis utilizing a novel integration vector. Gene.

[23] Datsenko KA, Wanner BL (2000). One-step inactivation of chromosomal genes in *Escherichia coli* K-12 using PCR products. Proc Natl Acad Sci USA.

[24] Gust B, Chandra G, Jakimowicz D, Yuqing T, Bruton CJ, Chater KF (2004). Lambda red-mediated genetic manipulation of antibiotic-producing streptomyces. Adv Appl Microbiol.

[25] Paget MSB, Chamberlin L, Atrih A, Foster SJ, Buttner MJ (1999). Evidence that the extracytoplasmic function sigma factor sigma(e) is required for normal cell wall structure in *Streptomyces coelicolor* A3(2). J Bacteriol.

[26] Kieser T, Bibb MJ, Buttner MJ, Chater KF, Hopwood DA (2000). Practical streptomyces genetics.

[27] Weber T, Blin K, Duddela S, Krug D, Kim HU, Bruccoleri R, Lee SY, Fischbach MA, Müller R, Wohlleben W, Breitling R, Takano E, Medema MH (2015). antiSMASH 3.0—a comprehensive resource for the genome mining of biosynthetic gene clusters. Nucleic Acids Res.

[28] Caffrey P (2003). Conserved amino acid residues correlating with ketoreductase stereospecificity in modular polyketide synthases. ChemBioChem.

[29] Haydock SF, Aparicio JF, Molnar I, Schwecke T, Khaw LE, Konig A, Marsden AFA, Galloway IS, Staunton J, Leadlay PF (1995). Divergent sequence motifs correlated with the substrate-specificity of (methyl)malonyl-coa-acyl carrier protein transacylase domains in modular polyketide syntheses. FEBS Lett.

[30] Reeves CD, Murli S, Ashley GW, Piagentini M, Hutchinson CR, McDaniel R (2001). Alteration of the substrate specificity of a modular polyketide synthase acyltransferase domain through site-specific mutations. Biochemistry.

[31] Bevitt DJ, Cortes J, Haydock SF, Leadlay PF (1992). 6-Deoxyerythronolide-B synthase from *Saccharopolyspora erythraea*: cloning of the structural gene, sequence-analysis and inferred domain-structure of the multifunctional enzyme. Eur J Biochem.

[32] Nagano S, Cupp-Vickery JR, Poulos TL (2005). Crystal structures of the ferrous dioxygen complex of wild-type cytochrome P450eryF and its mutants, A245S and A245T: investigation of the proton transfer system in P450eryF. J Biol Chem.

[33] Parajuli N, Basnet DB, Chan Lee H, Sohng JK, Liou K (2004). Genome analyses of *Streptomyces peucetius* ATCC 27952 for the identification and comparison of cytochrome P450 complement with other Streptomyces. Arch Biochem Biophys.

[34] Tang GL, Cheng YQ, Shen B (2004). Leinamycin biosynthesis revealing unprecedented architectural complexity for a hybrid polyketide synthase and nonribosomal peptide synthetase. Chem Biol.

[35] He HY, Pan HX, Wu LF, Zhang BB, Chai HB, Liu W, Tang GL (2012). Quartromicin biosynthesis: two alternative polyketide chains produced by one polyketide synthase assembly line. Chem Biol.

[36] Chiu HT, Chen YL, Chen CY, Jin C, Lee MN, Lin YC (2009). Molecular cloning, sequence analysis and functional characterization of the gene cluster for biosynthesis of K-252a and its analogs. Mol BioSyst.

[37] Chen C, Liu X, Abdel-Mageed WM, Guo H, Hou W, Jaspars M, Li L, Xie F, Ren B, Wang Q, Dai H, Song F, Zhang L (2013). Nivetetracyclates A and B: novel compounds isolated from *Streptomyces niveus*. Org Lett.

[38] Kavanagh KL, Jörnvall H, Persson B, Oppermann U (2008). Medium- and short-chain dehydrogenase/reductase gene and protein families: the SDR superfamily: functional and structural diversity within a family of metabolic and regulatory enzymes. Cell Mol Life Sci.

[39] Mattheus W, Masschelein J, Gao LJ, Herdewijn P, Landuyt B, Volckaert G, Lavigne R (2010). The kalimantacin/batumin biosynthesis operon encodes a self-resistance isoform of the FabI bacterial target. Chem Biol.

[40] Walker JE, Saraste M, Runswick MJ, Gay NJ (1982). Distantlyrelated sequences in the alpha- and beta-subunits of ATP synthase, myosin, kinases and other ATP-requiring enzymes and a common nucleotide binding fold. EMBO J.

[41] Iyer LM, Leipe DD, Koonin EV, Aravind L (2004). Evolutionary history and higher order classification of AAA + ATPases. J Struct Biol.

[42] Barthelmebs L, Lecomte B, Diviès C, Cavin JF (2000). Inducible metabolism of phenolic acids in *Pediococcus pentosaceus* is encoded by an autoregulated operon which involves a new class of negative transcriptional regulator. J Bacteriol.

[43] Gury J, Barthelmebs L, Tran NP, Diviès C, Cavin JF (2004). Cloning, deletion, and characterization of PadR, the transcriptional repressor of the phenolic acid decarboxylase-encoding padA gene of *Lactobacillus plantarum*. Appl Environ Microbiol.

[44] De Silva RS, Kovacikova G, Lin W, Taylor RK, Skorupski K, Kull FJ (2005). Crystal structure of the virulence gene activator AphA from *Vibrio cholerae* reveals it is a novel member of the winged helix transcription factor superfamily. J Biol Chem.

[45] Madoori PK, Agustiandari H, Driessen AJ, Thunnissen AM (2009). Structure of the transcriptional regulator LmrR and its mechanism of multidrug recognition. EMBO J.

[46] Xu Z, Jakobi K, Welzel K, Hertweck C (2005). Biosynthesis of the antitumor agent chartreusin involves the oxidative rearrangement of an anthracyclic polyketide. Chem Biol.

[47] Blodgett JA, Oh DC, Cao S, Currie CR, Kolter R, Clardy J (2010). Common biosynthetic origins for polycyclic tetramate macrolactams from phylogenetically diverse bacteria. Proc Natl Acad Sci USA.

[48] Wu Q, Wu Z, Qu X, Liu W (2012). Insights into pyrroindomycin biosynthesis reveal a uniform paradigm for tetramate/tetronate formation. J Am Chem Soc.

[49] Sims JW, Schmidt EW (2008). Thioesterase-like role for fungal PKS-NRPS hybrid reductive domains. J Am Chem Soc.

[50] Liu X, Walsh CT (2009). Cyclopiazonic acid biosynthesis in *Aspergillus* sp.: characterization of a reductase-like R* domain in cyclopiazonate synthetase that forms and releases cyclo-acetoacetyl-l-tryptophan. Biochemistry.

[51] Zhang X, Zhong L, Du L, Chlipala GE, Lopez PC, Zhang W, Sherman DH, Li S (2016). Identification of an unexpected shunt pathway product provides new insightsinto tirandamycin biosynthesis. Tetrahedron Lett.

[52] Woo AJ, Strohl WR, Priestley ND (1999). Nonactin biosynthesis: the product of nonS catalyzes the formation of the furan ring of nonactic acid. Antimicrob Agents Chemother.

[53] Demydchuk Y, Sun Y, Hong H, Staunton J, Spencer JB, Leadlay PF (2008). Analysis of the tetronomycin gene cluster: insights into the biosynthesis of a polyether tetronate antibiotic. ChemBioChem.

[54] Bode HB, Zeeck A (2000). Biosynthesis of kendomycin: origin of the oxygen atoms and further investigations. J Chem Soc Perkin Trans.

[55] Richter ME, Traitcheva N, Knüpfer U, Hertweck C (2008). Sequential asymmetric polyketide heterocyclization catalyzed by a single cytochrome P450 monooxygenase (AurH). Angew Chem Int Ed.

