# Supplementary material for: Identification of nocamycin biosynthetic gene cluster from Saccharothrix syringae NRRL B-16468 and generation of new nocamycin derivatives by manipulating gene cluster
Source: Microb Cell Fact. 2017 Jun 9;16:100. doi: 10.1186/s12934-017-0718-5 (PMC5466765; doi:10.1186/s12934-017-0718-5)
Supplement: Supplementary file 1 — Additional file 1: Table S1. Conserved motifs from PKS modules in nocamycin gene cluster. Figure S1. Inactivation of ncmB by gene disruption. Figure S2. Inactivation of NcmL by gene disruption. Figure S3. Inactivation of NcmG by gene disruption. Figure S4. Multiple sequence alignment of NcmC and its homologous protein sequence. Figure S5. Multiple sequence alignments of the cytochrome P450 domains of NcmG and NcmO. Figure S6. Unrooted phylogenetical tree of NcmO and NcmG with TrdI, SlgO1, SlgO2 and other cytochrome P450s. Figure S7. Multiple sequence alignments of NcmL with confirmed proteins that contain biocovently linked FAD cofactor. Figure S8. HR–ESI–MS of nocamycin III (4). Figure S9. HR–ESI–MS of nocamycin IV (5). Figure S10. 1H NMR (700 MHz) spectrum of compound 4 in CDCl3. Figure S11. 13C NMR (176 MHz) spectrum of compound 4 in CDCl3. Figure S12. DEPT 135 spectrum of compound 4 in CDCl3. Figure S13. 1H–1H COSY spectrum of compound 4 in CDCl3. Figure S14. HSQC spectrum of compound 4 in CDCl3. Figure S15. HMBC spectrum of compound 4 in CDCl3. Figure S16. NOESY spectrum of compound 4 in CDCl3. Figure S17. 1H NMR (500 MHz) spectrum of compound 5 in MeOD. Figure S18. 13C NMR (125 MHz) spectrum of compound 5 in MeOD. Figure S19. 1H–1H COSY spectrum of compound 5 in MeOD. Figure S20. HSQC spectrum of compound 5 in MeOD. Figure S21. HMBC spectrum of compound 5 in MeOD. [file 12934_2017_718_MOESM1_ESM.docx]

***Microbial Cell Factory***

**Identification of nocamycin biosynthetic gene cluster from *Saccharothrix syringae* NRRL B-16468 and generation of new nocamycin derivatives by manipulating gene cluster**

Xuhua Mo^1^*, Chunrong Shi^1^, Chun Gui^2^, Yanjiao Zhang^1^, Jianhua Ju^2^, Qingji Wang^1^*

Correspondence: [xhmo2013@163.com](mailto:xhmo2013@163.com); qingjiwang2016@hotmail.com

^1^Shandong Key Laboratory of Applied Mycology, School of Life Sciences, Qingdao Agricultural University, Qingdao 266109, China.

Table S1. Conserved motifs from PKS modules in nocamycin gene cluster.

| **Module** | **KS Motif** | **AT Motif** | **ACP Motif** | **KR Motif** | **DH Motif** |
| --- | --- | --- | --- | --- | --- |
| M0 | DTAQSSALV | GHSVGE…HAFH | LGFDSL |  |  |
| M1 | DTACSSSLV | GHSQGE…YASH | QGFDSL | HTA××LDD...L...AY |  |
| M2 | DTACSSSLV | GHSQGE…YASH | LGVDSL |  |  |
| M3  M4  M5 | DTACSSSLV  DTACSSSLV  DTACSSSLV | GHSVGE…HAFH  GHSQGE…YASH  GHSQGE…YASH | LGFDSL  LGLDSL  LGFDSL | HTA××VDD...L...NY  HTA××LAD...L...NY  HAA××IGL...L...AY | H×××G××××P |
| M6 | DTACSASLT | GHSQGE…YASH | LGFESL | HVA××VDD...L..NY | H×××G××××P |
| M7 | DTACSSSLV | GHSVGE…HAFH | IGFDSL | HVA××VDD...F...AY | H×××G××××P |
| M8 | DTACSSSLV | GHSVGE…HAFH | LGFASL |  |  |
| conserved motif | DTACSSSLV  Aparicio et al. (1996) | G×S×GE... HAFH^a^  G×S×GE... YASH^b^  Haydock et al. (1995)  Reeves et al. (2001) | LG×DSL  Wakil et al. (1989) | H×A....W...Y^c^  Caffrey et al.(2003) | H×××G××××P  Bevitt et al.(1992) |

note：KS: ketosynthase, AT: acyl transferase, ACP: acyl carrier protein, DH:dehydratase, KR: ketoreductase,

^a^Malonyl-CoA specific motif , ^b^methylmalonyl-CoA specific motif, ^c^A-type KR motif, ^d^B-type KR motif.


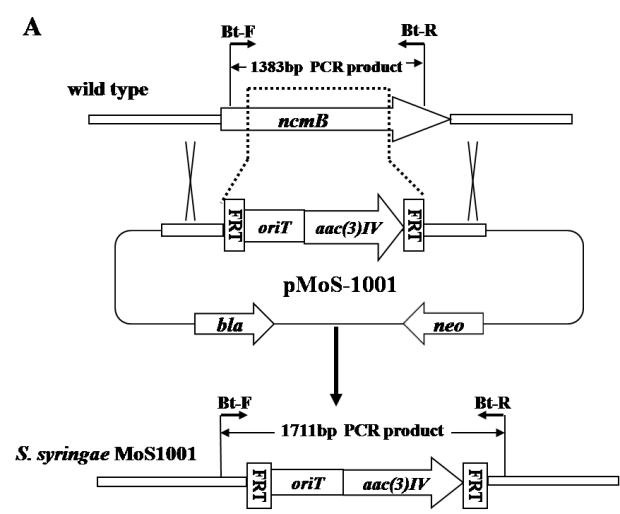

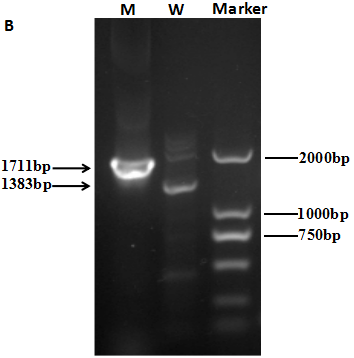


**Figure S1**. Inactivation of *ncmB* by gene disruption. (A) Construction of *ncmB* gene replacement mutant *S. syringae* MoS1001 and PCR fragement length. (B) PCR analysis of the double-crossover mutant. W: *S. syringae* MoS1001 wild type (1383 bp); M: mutant strain *S. syringae* MoS1001 (1711 bp); Marker: DNA molecular ladder.


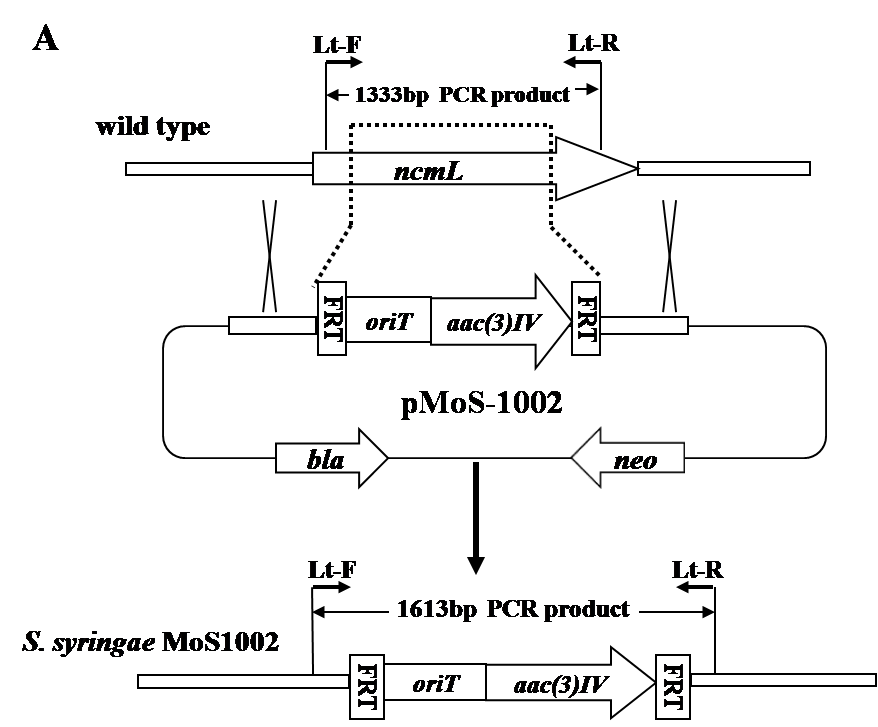

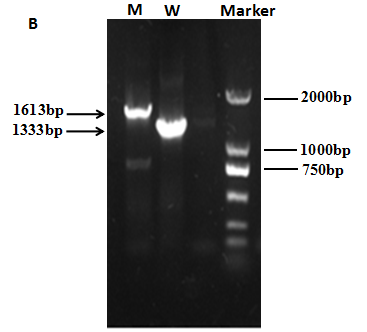


**Figure S2**. Inactivation of *NcmL* by gene disruption. (A) Construction of *ncmL* gene replacement mutant *S. syringae* MoS1002 and PCR fragement length. (B) PCR analysis of the double-crossover mutant. W: *S. syringae* MoS1002 wild type (1333 bp); M: mutant strain *S. syringae* MoS1002 (1613 bp); Marker: DNA molecular ladder.


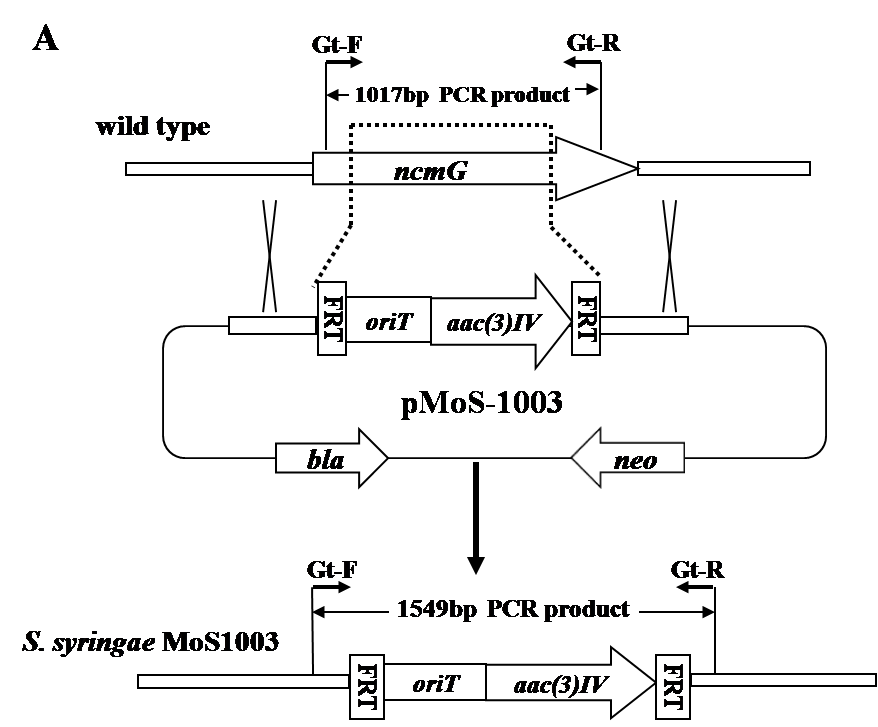


**Figure S3**. Inactivation of *NcmG* by gene disruption. (A) Construction of *ncmG* gene replacement mutant *S. syringae* MoS1003 and PCR fragement length. (B) PCR analysis of the double-crossover mutant. W: *S. syringae* MoS1003 wild type (1017 bp); M: mutant strain *S. syringae* MoS1003 (1549 bp); Marker: DNA molecular ladder.

LipX2 -------MTVQQPLATTAPPTWKVLNEGGSAEPVVLAVDFAVSGRPESTFSDLGRLLA--

SlgL MNTSLNASDAPRAAAPAAPDGWKVLHDGGPGE-LVLAVDYASTGRRESSFFDIVPNLP--

KirHI ---------------MPSPSVWNVVLDAASDADVVLATDFPVTGRNEGGFADLTPSLG--

TrdC ---------------MTDPQTWR-VLGNADAQELVLAVDFDATGRAEGRFTDLASGLGDL

NcmC ---------------MTAPRAWRPIAGGPPAGPLVLAVDFAATGRPEAAFADLVARLD--

. * *. : :***.*: :** *. * *: *

★

LipX2 RGVPLWETRQPEPEQARTFGGEDFASYWVRGVRDTGRPVRAVLGYCVGGLYAARVAQLLA

SlgL ADRTVWETTQPALGQETEMGGAAYVARWMRAVTESGRYVRAVMGYCVGSVFAAALAEEIG

KirHI LDCALWQTVAPARAPGPGIDPDEYLAPWLAEVAASGRRVRAVLGYCAGSVFAGALAERIA

TrdC LPCSIWETIP--QEAGTGAGDADPVERWLEEVAADGRPVRAVFGFCVGGVYAGAIAQRLA

NcmC PGTEVWESLQPPLGTETGMVAEDYVTRWEEEVRASGRRIGAVLGFCAGSAFAGELAVRLA

:*:: * * ** : **:*:*.*. :*. :* :.

★

LipX2 AEQDEAPAVVLFDPEPPHRAGMVADFRAAVARLSSVLSPEESALAGQAADEAAQQSA-GI

SlgL RIQAEPPAVLMFDPSVPVSVNLRHDFCNVIDHYATILTEDEVVHCKAEAHRLFAEDD-DF

KirHI DRQPVAPQVVVFDPELVDVPTVHLQFGRVIGNMTAVLTPAEVDELAGAAERLAARPGITP

TrdC ARQEQAPALVLFDPEQSGPLTLYSQFHKVLDSMSALLSAEETAQAADEARR-AHEQHTTM

NcmC RSQPRSPRLVVFDPESPTTSTLYYQFRKVVESLAGVLGEQAAREALAEGTAAADRIG-DV

* .* :::***. : :* .: : :* . .

LipX2 AALGAALTEVFTGIAGAAFDRAGLPAGLADELSGAYAAFVAYIAAAAAFDPAVSWAGATA

SlgL DRLGARYSRMFEEVVETAFVRSELDVELGRELSDTFVSFVRYLVAARQVDPSRVWARGTA

KirHI AAFATELYAIFEPIGSAALRRAGLDEGYAGELVALVGSFMTYLGVAAGLDPRPGWSRATV

TrdC AALGAELLDVYRRTAKPAFDRLELDDRRTEEITAVFASFVGYLIAAADIDPLPAWREAVV

NcmC EGLGAELVRVFTAAGRAACAAADLDDEFADELTATYRSFVSYLVAAAAVDHVKCWSGAVA

:.: :: .* * *: :*: *: .* .* * ...

★

LipX2 LTTPAADP-----------HARYARRLIPVDAAHDDILRSPLTADVLTGLLTRSRPQG-

SlgL VSSRDSTP-----------AADLAGREIQFDIDHLDILRDPGVGRAVTELLTAPR----

KirHI VTSASPASGLNRMRATPGLAPIAVADEVRFEVEHRDLLRTPEVATTVAGLLGTGSQARR

TrdC VTSSSPTSGLNGSRAAG--QDIAVGRELRVDVPHVDLLRDPGVTRTVTELLGADRPA--

NcmC VSSATPTSGLNPLDPAA--RAALVERELTFDVHHADLLRDPGVARAVARLLA-------

::: . . . : .: * *:** * . .:: **

**Figure S4**. Multiple sequence alignments of NcmC and its homologous protein sequence TrdC, SlgL, LipX2, KirI by using CLUSTALX (1.81). The conserved catalytic site are marked in red color.

**oxygen binding region**

NcmG **211** ARDEGDERLTEEELVVFGVTLLLAGLETTANQIGNFAYHLLVRPGRYAALAADPAGVPAA

NcmO RNAE-AGTLEHDEVVGMAAFLLISGFETTANMISMGTIGLLENPDQLALLREDPARAAGA

TrdI ARDV-DDRLTETELVSMAFLLFIGGHETTVNTLGNGTLHLMRNLDQWEALRQDRSLLPGA

SlgO1 ARDD-EDRLSETELLSMTFLLLVAGHETTVNTLGNGVFHLMADRDQWEKLLADRSLLPTA

SlgO2 ARDE-GDRLADSELVPMAFLLLVGGHETTTNLIGNGTLHLLRDRTKLRALLDDPALLPNA

Mur7 ACDE-EERLSEQELVSFAVTLLLAGHETTTDELGNFLYTLLVNPAHREQLRARPHMLGTA

LnmA ARDD-DDRLTEDELITFGVTLLVAGHETSAHQLGNMVYALLTHEDQLSLLREQPELLPRA

QmnO NHLE-KGEVTRENLLATILLLLNAGHETTANMISLGTLALLENPDQLAALRADPELVGSA

: ::: *: .* **:.. :. *: : * *

**k-helix**

NcmG **271** VEELLRYT-PIATTAGFTRVATEDVELGGVAVRAGEAVLVDLDSANRDEDVFAGAEELRL

NcmO VEELLRYFSVSDPAG--SRVALEDVAIGGTVIPAGSGVIALAGAANWDERVFPEPERLDI

TrdI VEEFLRLESPLKHAT--FRCATEDLRIGDTAIPAGDFVLLALASANRDPERFGDPHTLDV

SlgO1 VEEFLRIEGPLKHAT--FRCATECVRIGDVDIPAGDFVLLSLASANRDPRRFTRPHDLDV

SlgO2 VEEFLRFEGPIKHAT--FRYTTDEVEVDGVRIPAGELVLVSLVSANRDGERFTDPDRLDL

Mur7 IEELLRFV-PIGTLSGFTRIATEDVHLSGGLVRAGDAVVVQADSANRDESVFADPDELDF

LnmA VEELLRFV-PLGNGVGNARIALEDVELSGGTVRAGEGVVAAAVNANRDPRAFDDPDRLDI

QmnO VEEMLRYLSIGDIVP--ARITTEDLEVGGTTIKAGEGLIALLGAADWDPEVFPSPEVFDI

:**:** * : : : :.. : **. :: *: * * .. : .

**heme binding pokect**

NcmG **330** DRADNPHLAFGHGPHYCLGAGLARLELDVALRALLGGFPTLRLAVPAADLRWHTAKVVRG

NcmO TRDARAHLAFGHGAHQCIGLHLARLELEVVFGTLFRRVPTLRLTTPAEDLRYKEHANIYG

TrdI RRPTGGHVAFGHGIHYCLGAPLARMEAQVAFGVLLDTFPAMRLAVDPEDMRWRTSTLIRG

SlgO1 SRATGGHLAFGHGIHHCLGAPLARLEARIAFDALLQRFPGMELAVAPGELRWRSSTLIRG

SlgO2 TRAPGGNLAFGHGIHYCVGAPLARLEAQIAFRQLLERYPDMELAAEPAELYWRASTLMRG

Mur7 QREPNRHLAFGYGPHHCLGAQLARIELRAAIGALLTRLPALALAVPEHEVPWKLGRSALG

LnmA TREKNPHLAFGHGAHYCLGAQLARMELRVAIGGLLERFPGLRLAVPADQVEWKTGGLFRG

QmnO RRGARHHIAFGYGVHQCVGQNLARLELEILFRELVSRIPTLRLAAPVEDLPYKRQGAVYG

* ::***:* * *:* ***:* : *. * : *:. :: :: *

**Figure S5**. Multiple sequence alignments of the cytochrome P450 domains of NcmG and NcmO. The conserved motif are marked in red color.


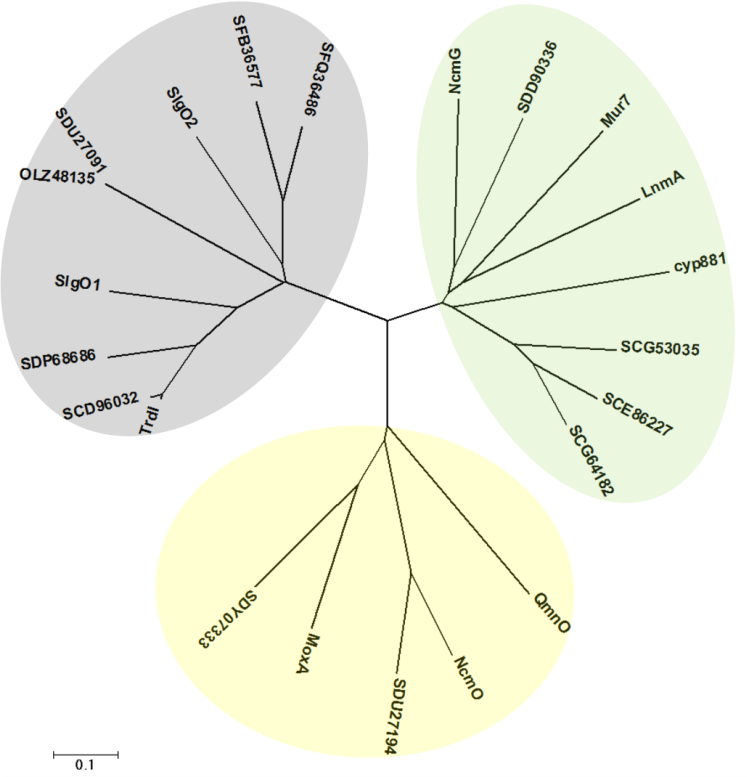


**Figure S6**. Unrooted phylogenetical tree of NcmO and NcmG with TrdI, SlgO1, SlgO2 and other cytochrome P450s.

NcmL 40 -SKPSTASRGKAGIQPRSLEVFDDLGVVDEVVAAG-TQRLPFRRFTRDRLVGEAIPYLHH

TamL/TrdL PGDIRYEDLRR-GENLRFVGDPEEIHLVGSAAEIEQVLSRAVRSGKRVAVRSGGHCYEDF

BusJ PGDPRYPDLVV-GHNPRFTGKPERIHIAGSTEDVVHAVAEAVRTGRRVGVRSGGHCFENL

SpnJ PDDPRYPDLVV-GHNPRFTGKPERIHIASSAEDVVHAVADAVRTGRRVGVRSGGHCFENL

AknOx RVDRRYQDLVTRGFNGRFRGRPDVVYVVHTADQVVDAVNQAMAAGQRIAVRSGGHCFEGF

BBE1 FNRFLHLSIQNPLFQNSLISKPSAIILPGSKEELSNTIRCIRKGSWTIRLRSGGHSYEGL

. : . : : . : . . :

NcmL 158 RSVTADYLVGCDGGRSTVRKALDLPFRGHTETDRRLLVGD---------VEVDGLEPDAW

TamL/TrdL CPDVGAGGHILGGGYGPLSRMHGSIVDYLHAVEVVVVDASGDARTVIATREPSDPNHDLW

BusJ CPGVGAGGHIPGGGYGPLSRRFGSVVDYLQGVEVVVVDRAGEVHIVEVDRNSIGAGHDLW

SpnJ CPGVGAGGHILGGGYGPLSRRFGSVVDYLQGVEVVVVDQAGEVHIVEADRNSTGAGHDLW

AknOx CPQVGVGGHVLGGGYGPLSRRDGVVADHLYAVEVVVVDASGRARKVVATSAADDPNRELW

BBE1 CPTVGTGGHISGGGFGMMSRKYGLAADN--VVDAILIDANG------AILDRQAMGEDVF

. .. .** . : : . .: :: : :

Figure S7. Multiple sequence alignment of NcmL with confirmed proteins that contain biocovently linked FAD cofactor. The conserved dual sites (H and C) for bicovalent attachment to FAD are marked in red color.

**A**


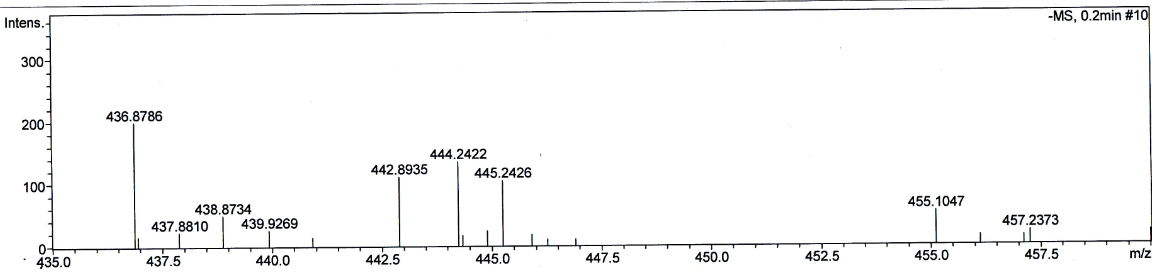


**B**


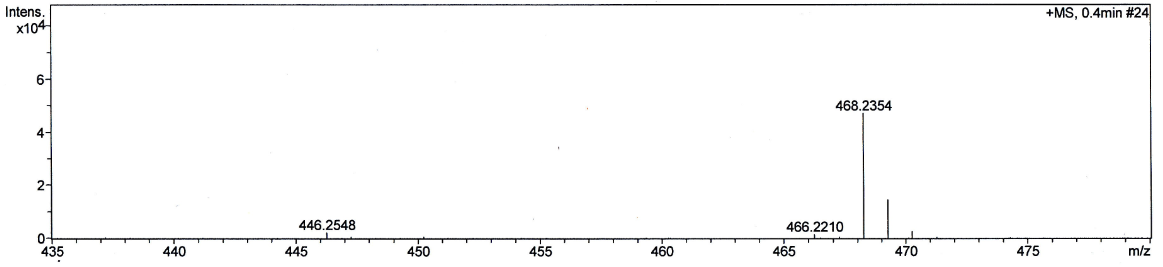


**Figure S8**. HR-ESI-MS of nocamycin III (**4**). (A) negative ion mold, [M-H]^-^ m/z=444.2422; (B) positive ion mold, [M+H]^+^ m/z=446.2548, [M+Na]^+^  m/z=468.2354.

**A**


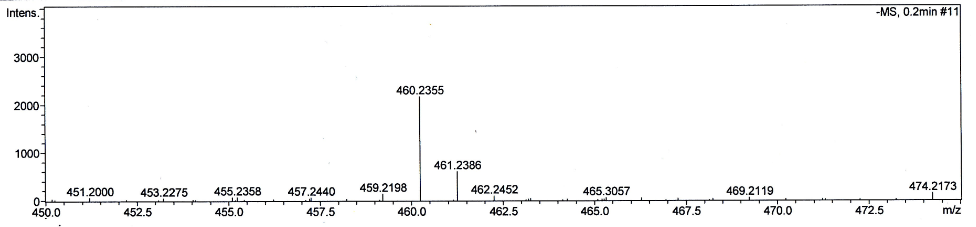


**B**


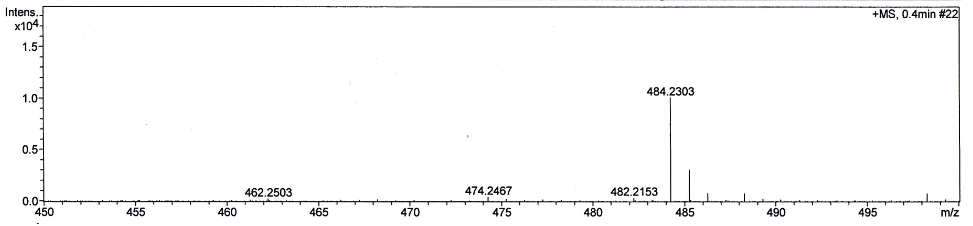


**Figure S9**. HR-ESI-MS of nocamycin IV (**5**). (A) negative ion mold, [M-H]^-^ m/z=460.2355; (B) positive ion mold, [M+H]^+^ m/z=462.2503, [M+Na]^+^ m/z=484.2303.


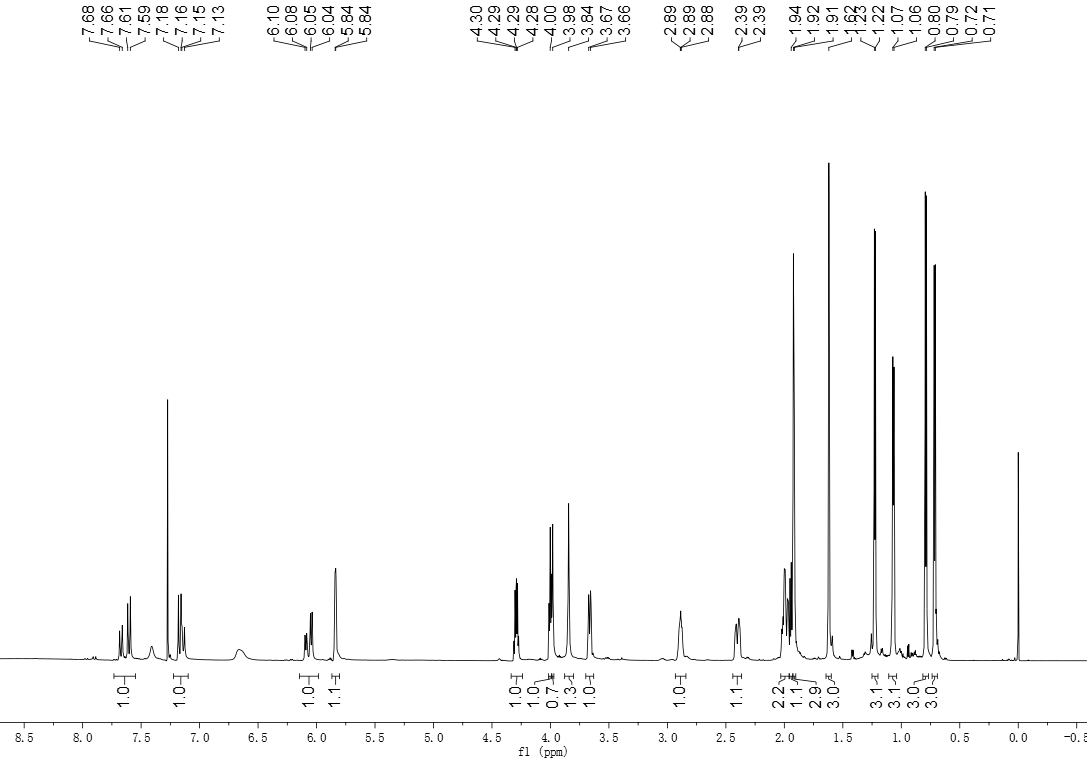


**Figure S10.**^1^H NMR (700 MHz) spectrum of compound **4** in CDCl_3_


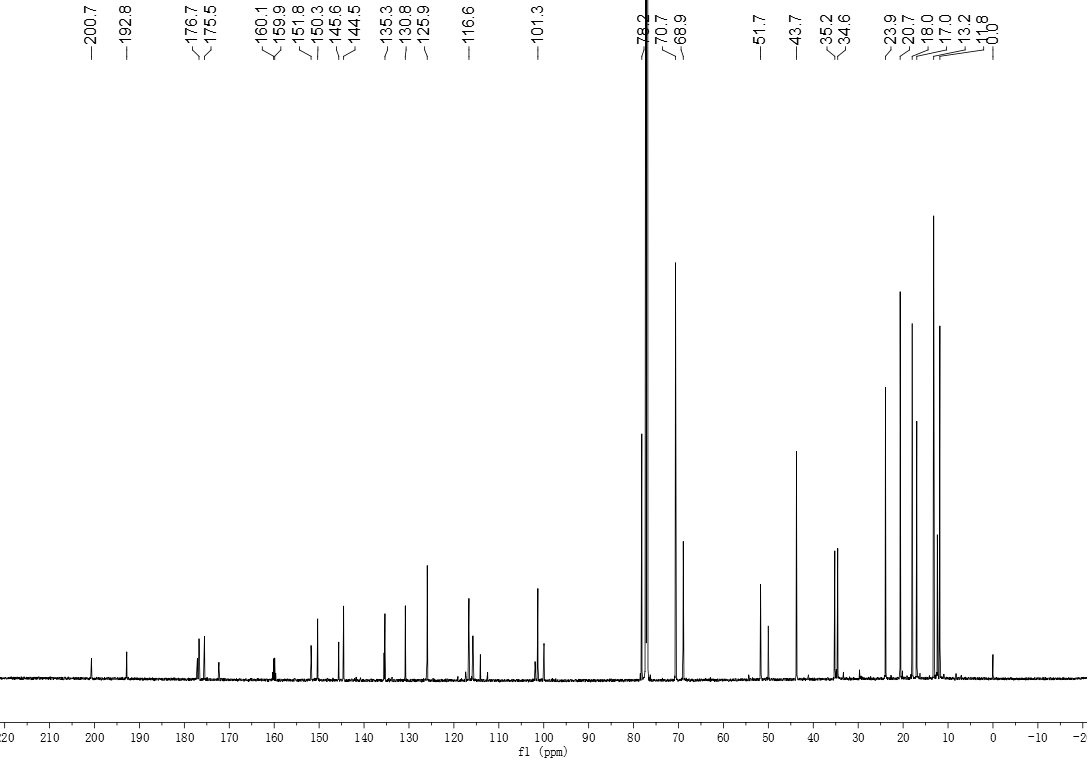
**Figure S11.** ^13^C NMR (176MHz) spectrum of compound **4** in CDCl_3_


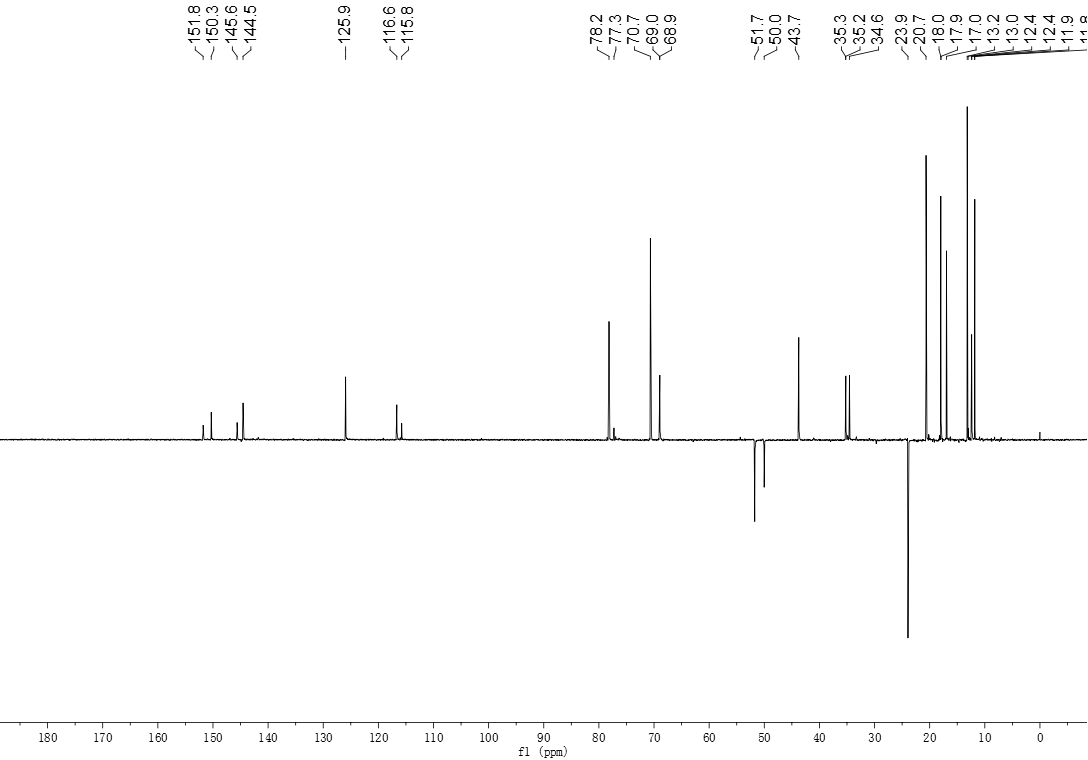


**Figure S12.** DEPT 135spectrum of compound **4** in CDCl_3_


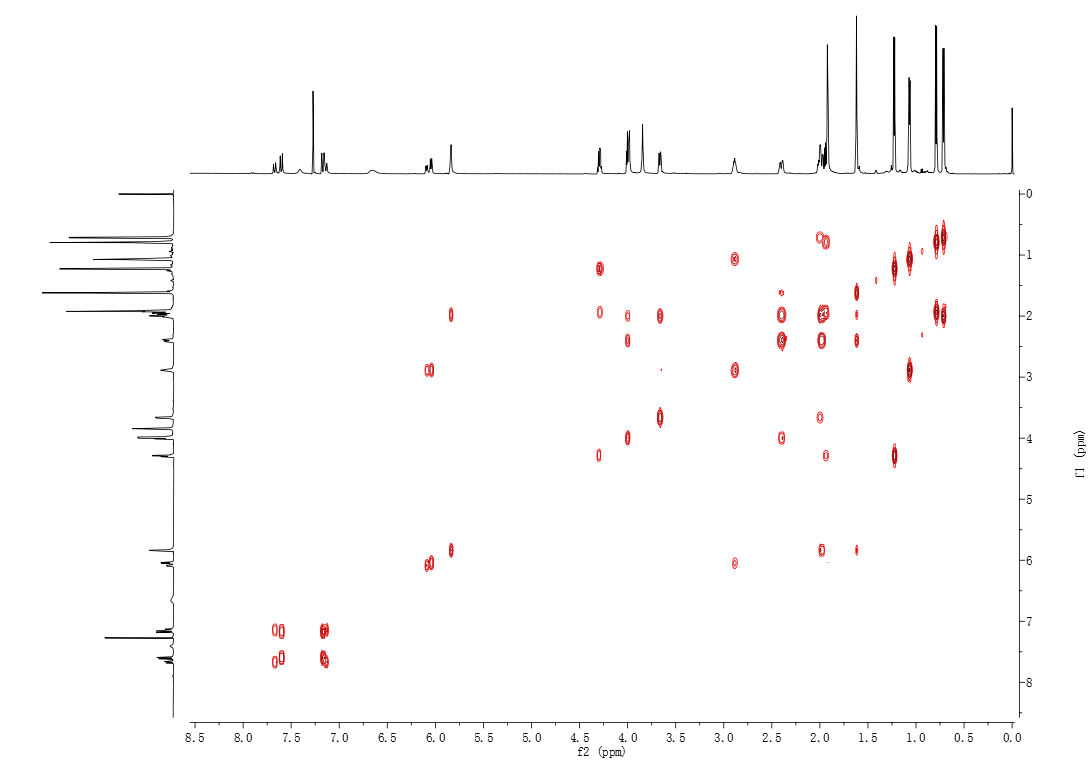


**Figure S13.** ^1^H-^1^H COSY spectrum of compound **4** in CDCl_3_


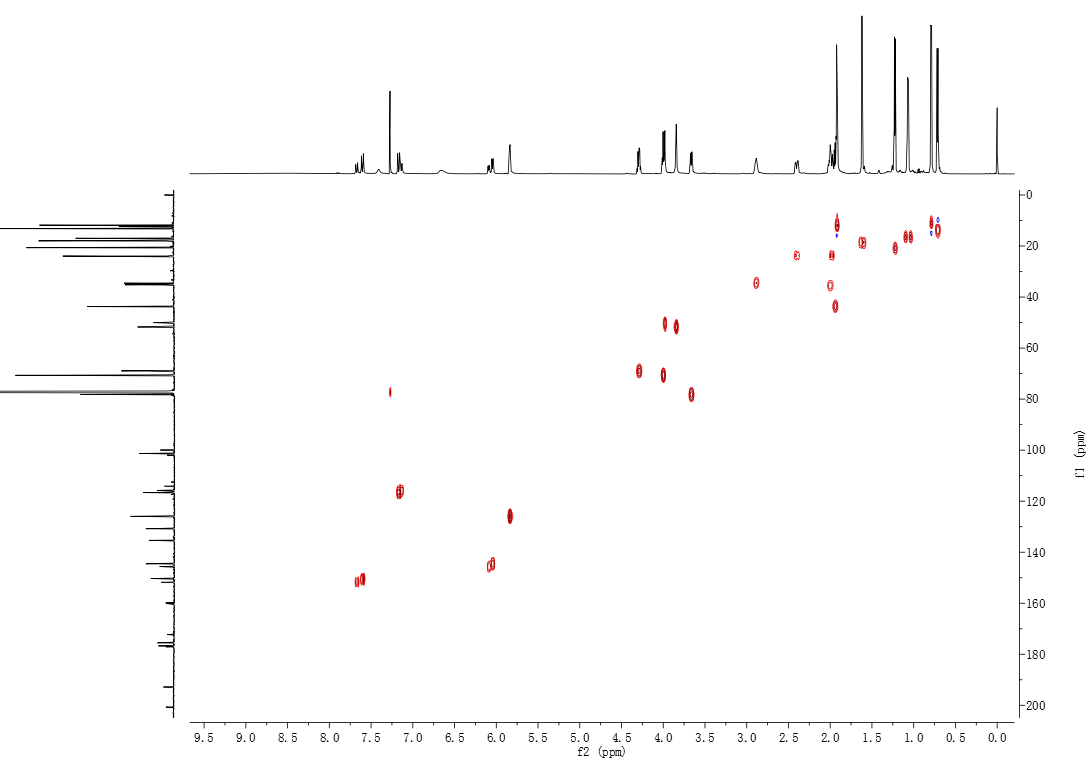


**Figure S14.** HSQC spectrum of compound **4** in CDCl_3_


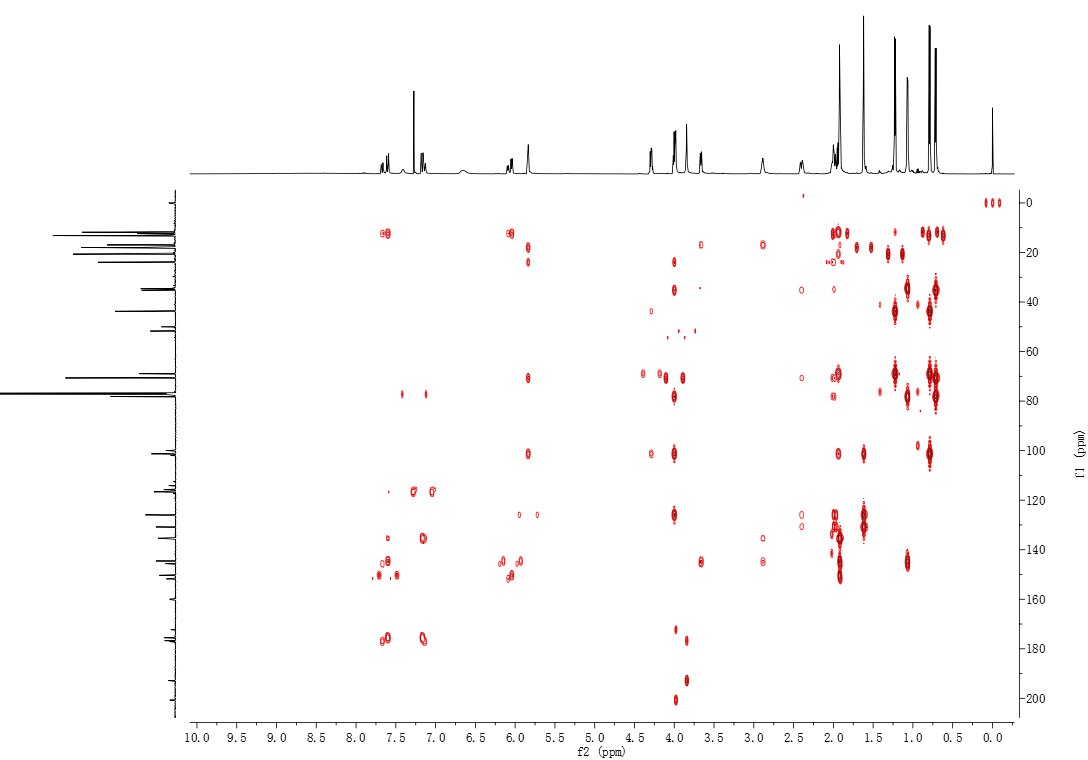


**Figure S15.** HMBC spectrum of compound **4** in CDCl_3_


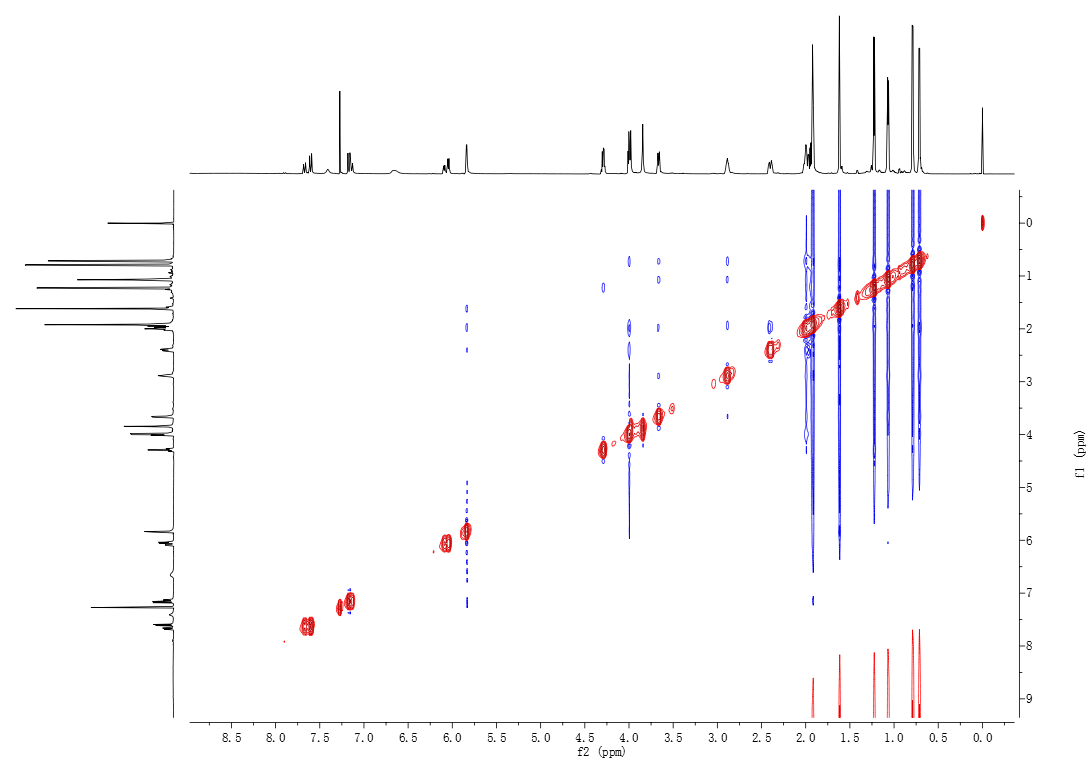


**Figure S16.** NOESYspectrum of compound **4** in CDCl_3_


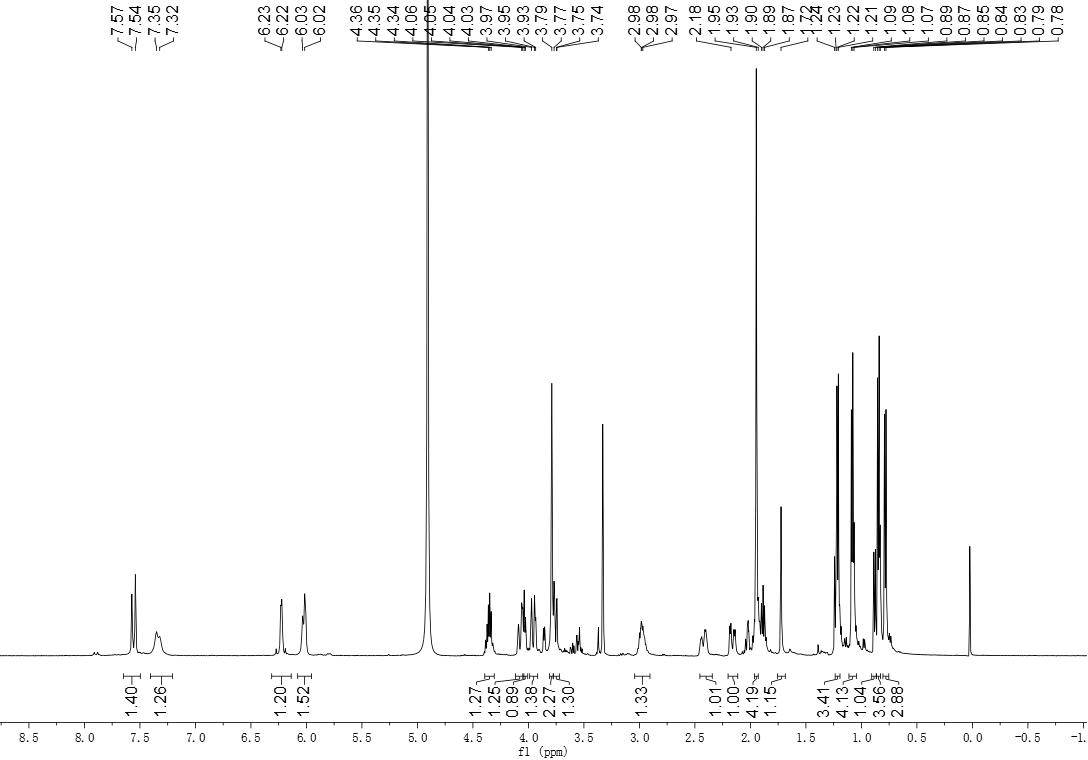


**Figure S17.** ^1^H NMR (500 MHz) spectrum of compound **5** in MeOD


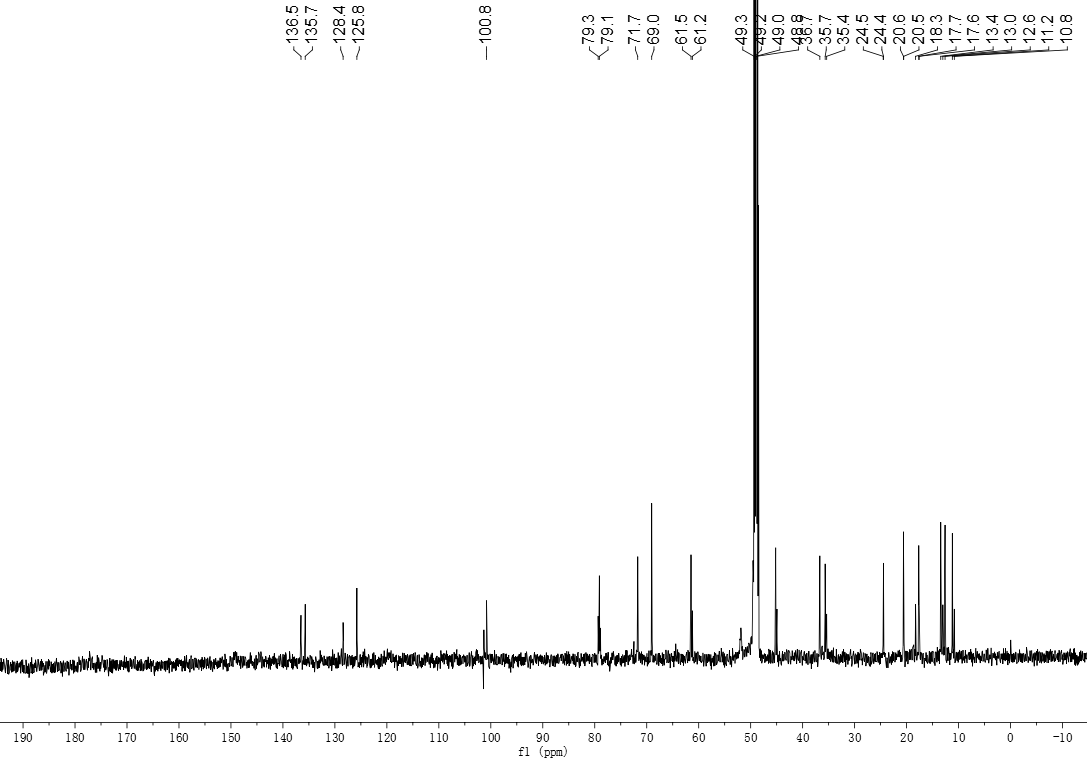


**Figure S18.** ^13^C NMR (125MHz) spectrum of compound **5** in MeOD


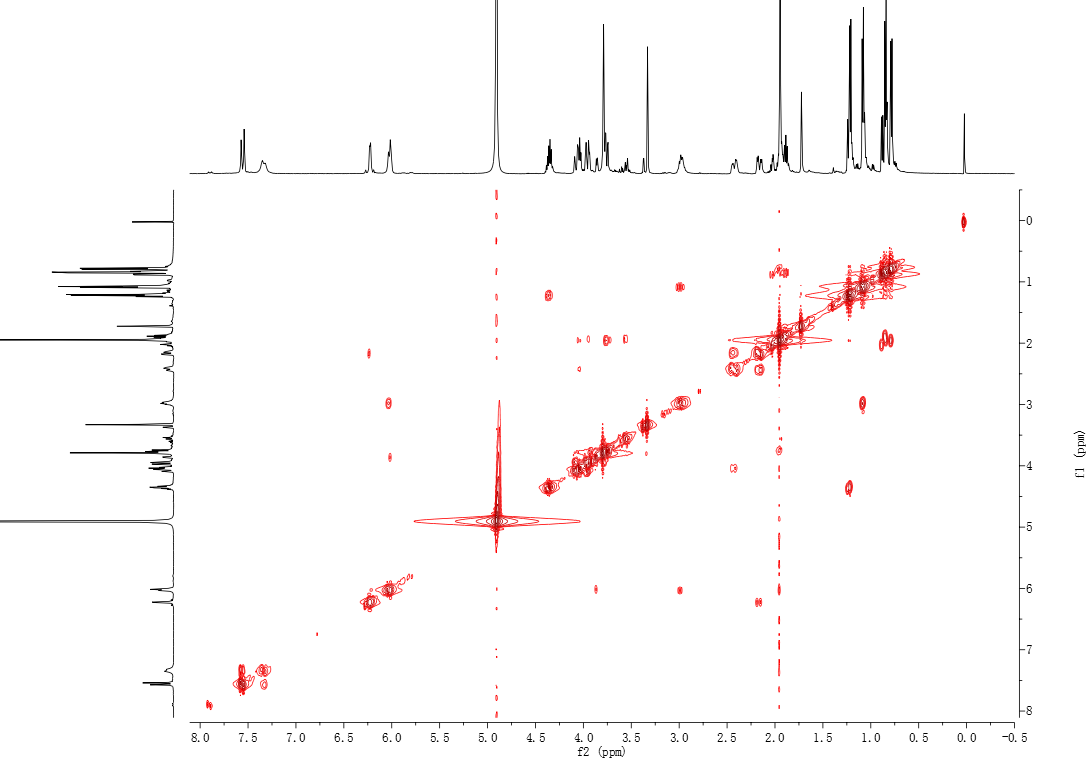


**Figure S19.** ^1^H-^1^H COSY spectrum of compound **5** in MeOD


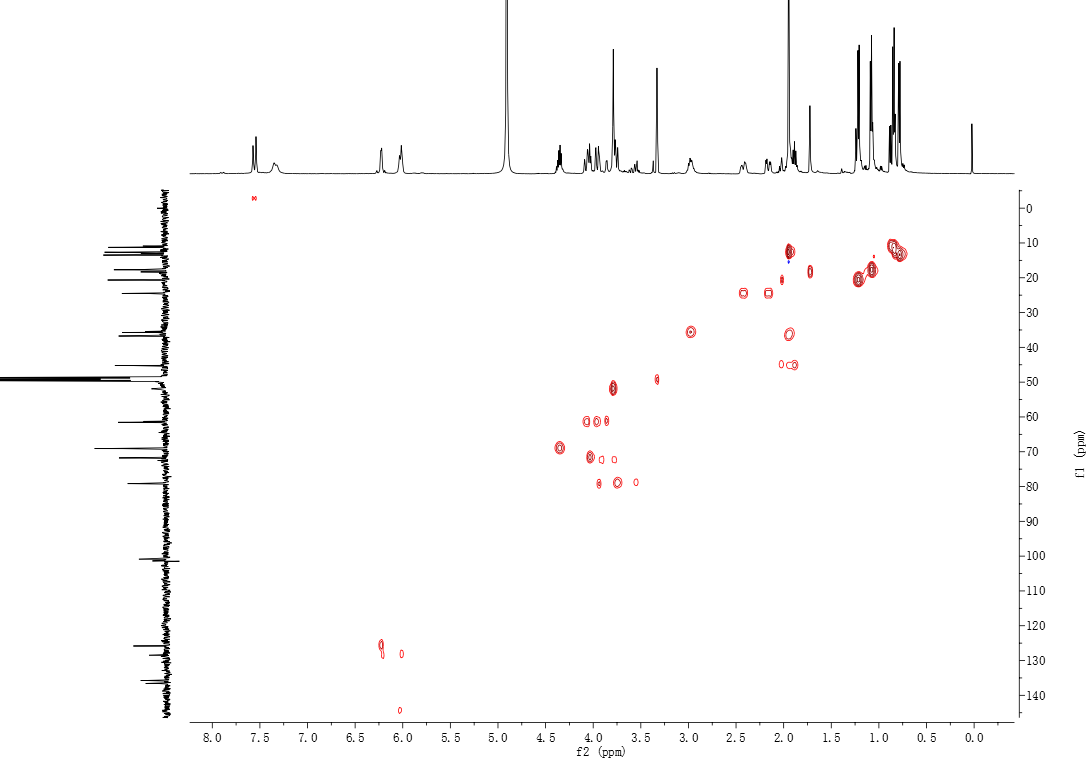


**Figure S20.** HSQC spectrum of compound **5** in MeOD


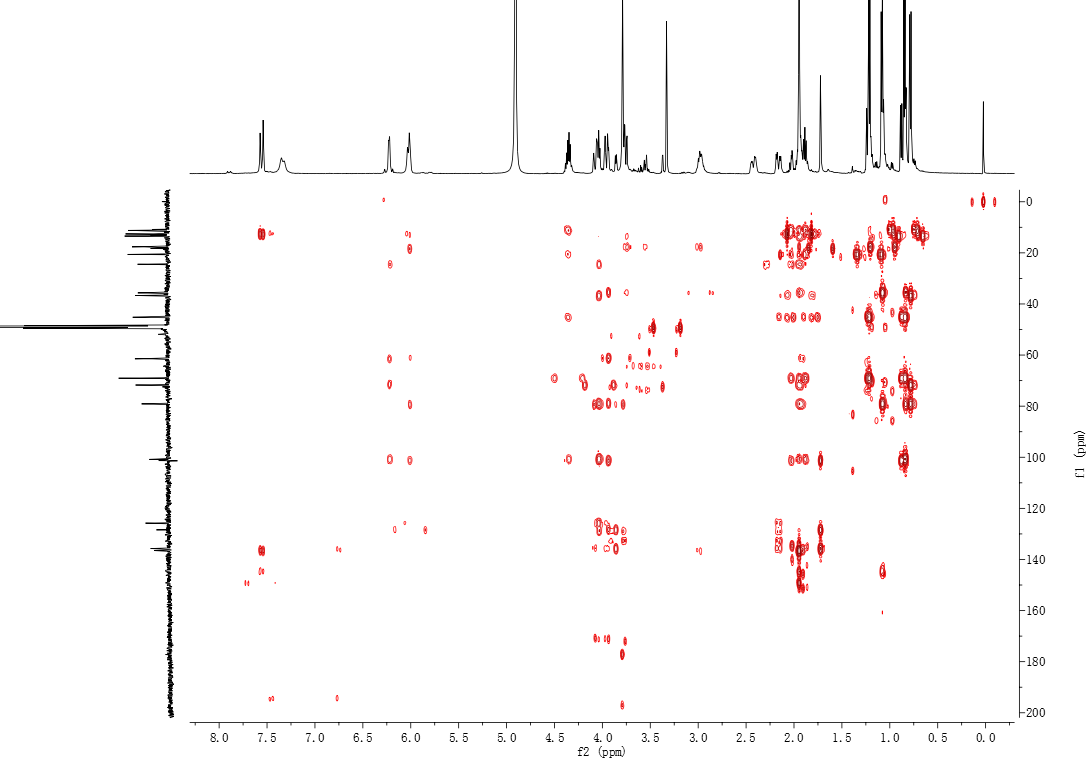


**Figure S21.** HMBC spectrum of compound **5** in MeOD
